# Vulnerability of macronutrients to the concurrent effects of enhanced temperature and atmospheric pCO_2_ in representative shelf sea sediment habitats

**DOI:** 10.1007/s10533-017-0340-y

**Published:** 2017-06-09

**Authors:** Jasmin A. Godbold, Rachel Hale, Christina L. Wood, Martin Solan

**Affiliations:** 1 Ocean and Earth Science, National Oceanography Centre Southampton, University of Southampton, European Way, Southampton, SO14 3ZH UK; 2 Biological Sciences, University of Southampton, Highfield Campus, Southampton, SO17 1BJ UK

**Keywords:** Biodiversity, Ocean acidification, Multiple stressors, Organic carbon, Macronutrients, Bioturbation

## Abstract

Fundamental changes in seawater carbonate chemistry and sea surface temperatures associated with the ocean uptake of anthropogenic CO_2_ are accelerating, but investigations of the susceptibility of biogeochemical processes to the simultaneous occurrence of multiple components of climate change are uncommon. Here, we quantify how concurrent changes in enhanced temperature and atmospheric pCO_2_, coupled with an associated shift in macrofaunal community structure and behavior (sediment particle reworking and bioirrigation), modify net carbon and nutrient concentrations (NH_4_-N, NO_x_-N, PO_4_-P) in representative shelf sea sediment habitats (mud, sandy-mud, muddy-sand and sand) of the Celtic Sea. We show that net concentrations of organic carbon, nitrogen and phosphate are, irrespective of sediment type, largely unaffected by a simultaneous increase in temperature and atmospheric pCO_2_. However, our analyses also reveal that a reduction in macrofaunal species richness and total abundance occurs under future environmental conditions, varies across a gradient of cohesive to non-cohesive sediments, and negatively moderates biogeochemical processes, in particular nitrification. Our findings indicate that future environmental conditions are unlikely to have strong direct effects on biogeochemical processes but, particularly in muddy sands, the abundance, activity, composition and functional role of invertebrate communities are likely to be altered in ways that will be sufficient to regulate the function of the microbial community and the availability of nutrients in shelf sea waters.

**Electronic supplementary material:**

The online version of this article (doi:10.1007/s10533-017-0340-y) contains supplementary material, which is available to authorized users.

## Introduction

Continental shelf sediments play an important role in the biogeochemical cycling of organic matter (Burdige 2006), but the potential consequences of future environmental conditions on the processes that underpin macronutrient (Voss et al. 2013) and carbon cycling (Chen and Borges 2009) have received little attention. Organic matter mineralization is a temperature sensitive, microbial driven process (Robador et al. 2009) in which both organic nitrogen and phosphorus are transformed into their respective inorganic forms and are available for primary production. In pelagic environments, nitrification—the microbial process in which ammonia (NH_3_) is oxidized to inorganic nitrite (NO_2_^−^) and nitrate (NO_3_^−^)—is inhibited at low pH (Huesemann et al. 2002; Beman et al. 2011; Kitidis et al. 2011), but whether the same holds true for nitrogen cycling in sediments is less well understood. Whilst nitrifying microbial communities and nitrification processes in the absence of large infauna appear to be resilient to the impacts of ocean acidification (Kitidis et al. 2011; Tait et al. 2013; Gazeau et al. 2014), nitrification rates in sediments containing active faunal burrow structures have been found to significantly reduce under low pH conditions (Laverock et al. 2013; Braeckman et al. 2014), which may ultimately limit pelagic nutrient availability (Hutchins et al. 2009). As there is tight coupling between nitrogen and other nutrient cycles, in particular phosphorous, silica and iron (Elser et al.,^2007^; Harpole et al. 2011), changes to nutrient stoichiometry are likely and may have consequences for local productivity (Downing 1997). Whilst the effects of pCO_2_ on phosphorous biogeochemistry have been found to be insignificant in pelagic systems (Tanaka et al. 2008), the fate of phosphorus cycling in benthic sediments under future climate conditions is unclear. PO_4_ can be bound in the sediment by adsorbing to ferric (oxy)hydroxides forming solid ferrous phosphates (Patrick and Khalid 1974) and mobilised either as a result of sulfate reduction (Roden and Edmonds 1997) or dissolving iron (Fe) minerals (Gachter and Muller 2003). In soils and sediments, the primary control of PO_4_ release is, however, redox condition, where PO_4_ is immobilized by oxidized Fe (Li et al. 2012).

Warming has the potential to increase sedimentary metabolic rates resulting in anoxic conditions in surface sediments that support PO_4_ release (Cowan and Boynton 1996), but relatively little is known about the potential independent or interactive effects of ocean acidification and warming (independent effects of temperature and pCO_2_, Bulling et al. 2010; effect of temperature but not pCO_2_, Godbold and Solan 2013) on nutrient generation per se. Current understanding is largely based on short-term empirical studies that have focussed on the impacts of either temperature or ocean acidification in isolation on biogechemical cycling, rather than recognising that increasing atmospheric CO_2_ levels drive both ocean warming and acidification simultaneously (IPCC 2014). Importantly, the combined effects of elevated temperature and pCO_2_ are unlikely to be additive (Przeslawski et al. 2008; Bulling et al. 2010) and, under some circumstances, can be antagonistic; in corals for example, warming has the potential to either offset the effects of ocean acidification (McCulloch et al. 2012) or worsen impacts through additive stress effects (Anthony et al. 2008; Rudolfo-Metalpa 2010), whilst in bivalves moderate warming can lessen the effects of acidification on calcification (Kroeker et al. 2014). Similarly, seasonal differences in temperature can either exacerbate or buffer the effects of acidification on invertebrate mediated nutrient generation (Godbold and Solan 2013). Hence, there are a range of physical and biological factors, including the characteristics of the sediment, community composition, and anthropogenic activity (Hedges and Keil 1995; Burdige 2006; Rocha 2008; Mayor et al. 2012; Laverock et al. 2013; Sciberras et al. 2017; Hale et al. 2017), that moderate microbial processes linked to biogeochemical pathways (Kitidis et al. 2017; Bates et al. 2013) that are yet to be fully embraced when considering the ecological effects of climatic variables.

Global climate change clearly has the potential to modify the composition of species assemblages, by promoting tolerant species and reducing sensitive species, as has been demonstrated along natural CO_2_ gradients, where community structure shifts from one containing calcifying organisms to a community dominated by non-calcifying species, with a concomitant reduction in local biodiversity and species abundance (Hall-Spencer et al. 2008; Hale et al. 2011; Johnson et al. 2014; Meadows et al. 2015; Crook et al. 2016; Gambi et al. 2016). Similarly, elevated temperatures can increase the metabolic rate of organisms within their thermal tolerance window (Pörtner and Farrell 2008), accelerating infaunal burrowing and ventilation activity (Ouellette et al. 2004). This is important because the burrowing and ventilation activities of infaunal organisms can have a significant impact on biogeochemical cycling in shelf sediment habitats (Lohrer et al. 2004; Laverock et al. 2011), by increasing the oxygen availability and redox conditions in the sediment and enhancing microbial process rates that affect sediment carbon and nutrient cycling (Jickells 1998; Johnson et al. 1999; Laverock et al. 2013). Organism responses to future climatic conditions are, however, highly variable and modified by both biological and environmental context (e.g. Rodolfo-Metalpa et al. 2011; Melzner et al. 2011; Comeau et al. 2010; Godbold and Solan 2013; Eklöf et al. 2015). Studies in which both temperature and ocean acidification have been simultaneously manipulated, suggest that communities are likely to be more affected by the impacts of warming than ocean acidification (e.g. Hale et al. 2011; Eklöf et al. 2015). On balance, however, recent reviews indicate a trend towards enhanced biological sensitivity to ocean acidification when taxa are simultaneously exposed to elevated temperatures and acidification (Kroeker et al. 2013).

Knowledge on the extent to which invertebrate community structure and specific taxa influence the mechanistic controls of biogeochemistry is scarce (Hansen et al. 1996; Botto et al. 2005; Gilbertson et al. 2012), especially in relation to climatic forcing (Tait et al. 2014). Nevertheless, the biological control of biogeochemical dynamics will be the net result of multiple direct and indirect species responses to forcing, yet few studies consider biological interactions (Alsterberg et al. 2013), sublethal effects on functioning (Sarmento et al. 2016), the role of different trophic levels (Hicks et al. 2011; Widdicombe et al. 2013) or the interdependencies between climatic drivers and geochemistry (Kroeker et al. 2014) that manifest over time (Godbold and Solan 2013). If we are to begin to close the disconnect that exists between the representation of climatic scenarios in experimental systems, the role of benthic invertebrates in biogeochemical models (Yool et al. 2013) and the context in which biodiversity-ecosystem process relations are altered under climatic forcing, there is a need to investigate the bulk response of the whole benthic system (Wernberg et al. 2012).

Here, we focus on the role of macrofaunal invertebrate communities in mediating carbon and nutrients across a gradient of representative shelf-sea sediments maintained under natural versus anticipated future climate regimes. Specifically, we determine the effects of whole community macrofaunal bioturbation on sediment organic carbon and net sediment–water nutrient concentrations. Our objective was to establish when macrofaunal traits and biogeochemical variables are closely coupled, and to provide insight as to whether organism-sediment relationships that underpin important aspects of shelf sea biogeochemistry will be sustained under future environmental conditions. We anticipated that the relative importance of the macrofaunal community and the abiotic environment in mediating biogeochemical processes would differ between distinct sediment environments, with biodiversity being most important and abiotic processes being least important in cohesive sediments and vice versa in non-cohesive sediments (Godbold and Solan 2009). Further, we speculated that in shelf sea systems where biogeochemical processes are closely controlled by faunal traits rather than high rates of sediment–water exchange, those habitats most susceptible to biogeochemical change would have (i) low functional redundancy (Micheli and Halpern 2005), and/or (ii) a disproportionate representation of species with enhanced sensitivity to the concurrent effects of elevated seawater temperature and altered pH associated with higher atmospheric pCO_2_ concentrations (Kroeker et al. 2013). Hence, our a priori hypothesis was that the importance of macrofauna in mediating sediment carbon and net sediment nutrient concentrations would be low in non-cohesive sediments where physical processes are predominant but high in cohesive sediments where diffusive processes are predominant. Further, the degree of species-environment coupling would be weakened under future environmental conditions.

## Materials and methods

To determine the vulnerability of macrofaunal coupled biogeochemical cycling to habitat type and anticipated future environmental conditions, we investigated whether macrofaunal community composition, behavior (sediment reworking and burrow ventilation), and sediment carbon and nutrients differed between field recovered intact sediment cores collected from four sites with contrasting sediment types (mud, sandy-mud, muddy-sand and sand; for detailed site characterization see Thompson et al. 2017) in the Celtic Sea (RRS Discovery, cruise DY008, March–April 2014). At each site, fifteen 0.08 m^2^ NIOZ (Netherlands Institute for Sea Research, Texel) cores were collected and subsampled to achieve intact sediment cores (internal dimensions, L × W × H: 20 × 20 × 12 cm). Five sediment cores from each site were preserved 6 days after recovery once community behavior and biogeochemical properties had been quantified. These cores were used to assess any differences in species composition between field and experimental communities maintained under laboratory conditions for extended periods of times (Range et al. 2014). The remaining ten sediment cores from each of the four sites were transferred to transparent acrylic aquaria (internal dimensions, L × W × H: 20 × 20 × 34 cm), overlain with 20 cm of seawater, continually aerated and maintained at 11 °C in the dark. At the end of the cruise, all aquaria were transferred to and maintained in the Biodiversity Ecosystem Futures Facility at the National Oceanography Centre, Southampton (University of Southampton).

### Experimental set-up and design

Aquaria were held in large water bath tanks in the dark, representative of bottom conditions in the Celtic Sea, and continually aerated by bubbling either ambient air or a treatment specific air-CO_2_ gas mixture through a glass pipette. Aquaria receiving ambient environmental conditions were held at 10.92 ± 0.40 °C (approximating the mean annual bottom temperature in the Celtic Sea, Thompson et al. 2017), and 391.07 ± 0.05 ppm atmospheric [CO_2_]. Aquaria receiving an environment representative of future climate conditions were held at ambient + 4 °C (14.56 ± 0.20 °C) and 1026 ± 0.24 ppm atmospheric [CO_2_], in line with future climate projections for 2100 (IPCC 2014). Our objective was to reflect the accepted view that [CO_2_] and temperature will rise over the long-term, rather than to make a specific prediction, allowing general trends in community attributes and biogeochemical processes to be defined. To avoid excessive accumulation of nutrients and metabolites, a partial (~50%) seawater change was performed on all aquaria once a week. Each sediment type (n = 4) × climate regime (n = 2) was replicated five times (total n = 40) and incubated for 189 days (~6 months).

Levels of [CO_2_] were controlled using a CO_2_-air mixing system (Godbold and Solan 2013), which maintained and monitored the supply of the air mixture to each aquarium using infrared gas analysers (Licor LI-840A, 1 per CO_2_ treatment). To minimize ambient air exchange, each aquarium was covered with a transparent acrylic lid (thickness, 1 mm). Following established protocol (Godbold and Solan 2013), pH (NBS scale, Mettler-Toledo InLab Expert Pro temperature–pH combination electrode), temperature and salinity (Mettler-Toledo InLab 737 IP67 temperature–conductivity combination electrode) were measured every 7 days and total alkalinity (A_T_) and nutrient concentrations (NH_4_-N, NO_x_-N, PO_4_-P) were measured every 14 days. Total alkalinity was analysed by titration (Apollo SciTech Alkalinity Titrator AS-ALK2) following standard protocols at the National Oceanography Centre, Southampton, UK Carbonate Facility. Concentrations of bicarbonate (H_2_CO_3_^−^), carbonate (CO_3_^2−^) and pCO_2_ were calculated from measured pH, A_T_, temperature and salinity (Dickson et al. 2007; Dickson 2010) using *CO*_*2*_ *calc* (Robbins et al. 2010) with appropriate solubility constants (Mehrbach et al. 1973, refit by Dickson and Millero 1987) and KSO_4_ (Dickson 1990) (Supplementary material Fig. S1).

### Measurements of sediment and water column condition

Seawater samples (30 mL, 0.45 μm pre-filtered, Nalgene) were taken after 5 days incubation to determine the concentration (μmol L^−1^) of ammonium (NH_4_-N), nitrite + nitrate (NO_x_-N) and phosphate (PO_4_-P) using a Tecator flow injection auto-analyser (FIA Star 5010 series). In addition, scrapes were taken from the sediment surface to determine sediment particle size (Malvern Mastersizer 2000, Supplementary material Fig. S2, Table S1) and percentage organic carbon content (loss on ignition, 375 °C, 1 h; OrgC %).

### Measurements of faunal behavior and community composition

Sediment particle reworking was estimated using fluorescent sediment profile imaging (f-SPI: Canon 400D, set to 10 s exposure, aperture f5.6 and ISO400, 3888 × 2592 pixels, effective resolution 88.47 μm per pixel). The redistribution of particulate sediment tracers (dyed sediment that fluoresces under ultraviolet light; 215 g aquarium^−1^, Brian Clegg Ltd., UK) is quantified from composite images (RGB colour, JPEG compression) of the four sides of each square aquarium after 6 days using a custom-made semi-automated macro that runs within ImageJ (Version 1.47a) (Solan et al. 2004a). From these data we calculated the mean (^f-SPI^L_mean_) and maximum (^f-SPI^L_max_) depths of particle redistribution. Following Hale et al. (2014) we also determined surface boundary roughness (SBR, the maximum vertical deviation of the sediment water interface) as an indicator of surficial activity. Burrow ventilation was estimated from absolute changes in the concentration of the inert tracer sodium bromide (Δ[Br^−^], mg L^−1^; negative values indicate increased ventilation activity; Forster et al. 1999) over a 4 h period on day 5, and determined using a Tecator flow injection auto-analyser (FIA Star 5010 series).

Macrofaunal community composition in the aquaria was determined by preserving the sediment in a 10% formalin (4% formaldehyde) solution buffered with seawater (salinity, 33) prior to sieving (500 μm). All taxa were identified to the lowest possible taxon (77% to species) and enumerated (total number of individuals present (abundance) and biomass). In addition, in order to identify shifts in functional trait composition between sediment types and climate regimes, organisms were separated into functional groups based on their reworking mode (Solan et al. 2004b; Queirós et al. 2013): surficial modifiers—organisms whose activities are mostly restricted to the uppermost layers (<1–2 cm) of the sediment profile; upward/downward conveyors—organisms that live vertically in the sediment and actively transport sediment from depth to the surface or, vice versa; biodiffusers—organisms whose activities result in constant, random sediment particle mixing over short distances that results in the progressive transport of particles throughout the depth of occupancy within the sediment profile.

### Statistical analyses

Analysis of variance (ANOVA) models were developed to investigate the interactive effects of sediment type and climate regime on each of the dependent variables: [NH_4_-N], [NO_x_-N], [PO_4_-P], OrgC, SBR, ^f-SPI^L_mean_, ^f-SPI^L_max,_ Δ[Br^−^], species richness. To assess whether there were any differences between field and experimental communities maintained under laboratory conditions (Range et al. 2014), we also developed a model that assessed the impact of experimental regime, i.e. communities in the aquaria maintained under ambient and future climate conditions for 6 months and those immediately preserved on recovery from the field (n = 5 site^−1^), and sediment type on species richness using ANOVA. Model assumptions were assessed visually (homogeneity: residuals vs fitted values, normality: QQ-plots, outliers/influential data points: Cooks-distance) and the optimal fixed effects structure was determined using backward selection informed by Akaike Information Criteria (AIC). Single and interactive treatment effects on macrofaunal community composition were visualised using two-dimensional non-metric multi-dimensional scaling (nMDS) based first on the abundance (square root transformed) of taxa and then their biomass. Community differences associated with sediment type and climate regime were determined using a permutational multivariate analysis of variance (PERMANOVA) with 999 iterations. The relative contribution of individual species driving community effects was identified using similarity percentages based on abundance or biomass (SIMPER). The multivariate assemblage composition data was square root transformed prior to the analyses, to reduce the disproportionate influence of numerically dominating species. As joint species absences were important to consider between treatments, the data were ‘zero-adjusted’ by adding a dummy variable of 1 for abundance and 0.0001 for biomass (Clarke et al. 2006). All analyses were conducted in R (version 3.1.2, R-development core team 2014) and the multivariate community data analyses were conducted using the ‘vegan’ package (Oksanen 2016).

## Results

As our experimental system manipulated atmospheric [CO_2_] to manipulate pH, we present a summary of the seawater carbonate chemistry in Supplementary Material (Fig. S1). Notably, we observed an increase in total alkalinity (A_T_) under future climate conditions relative to ambient climatic conditions, indicating that the processes affecting the carbonate system differed.

**Fig. 1:**
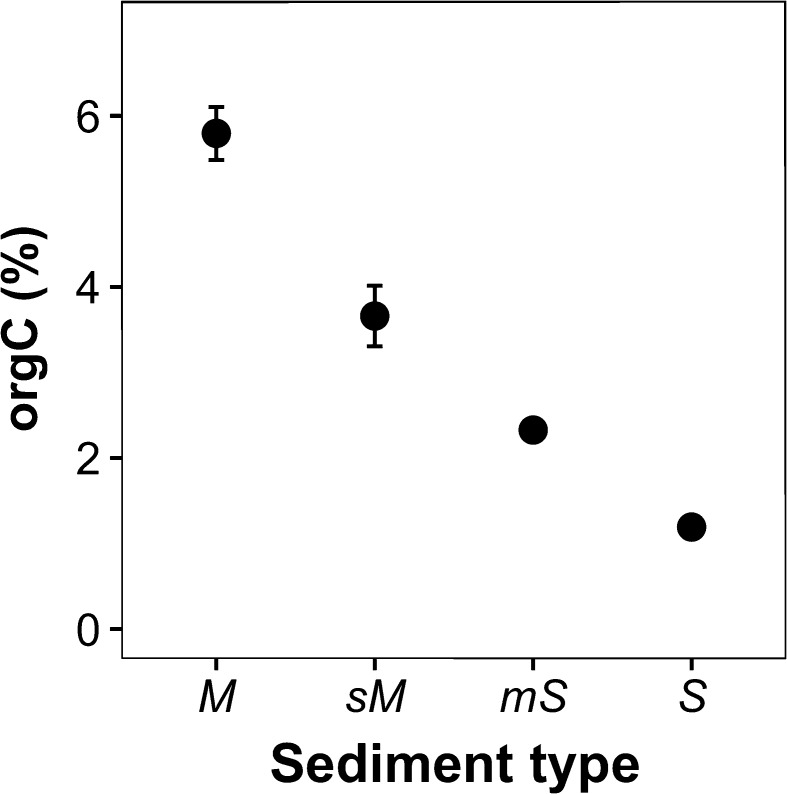
The effects of sediment type on mean (±SE, n = 10) total sediment organic carbon content (orgC, %) after 6 months. Sediment types are: *M* mud, *sM* sandy mud, *mS* muddy sand, *S* sand

Sediment total organic carbon content (OrgC, %) decreased with increasing grain size (ANOVA: F_3,35_ = 65.795, p < 0.0001), from 6.06% in muddy sediments to 1.26% in sandy sediments, but did not differ between climate regimes (ANOVA: F_1,35_ = 0.004, p = 0.949) (Fig. 1).

**Fig. 2:**
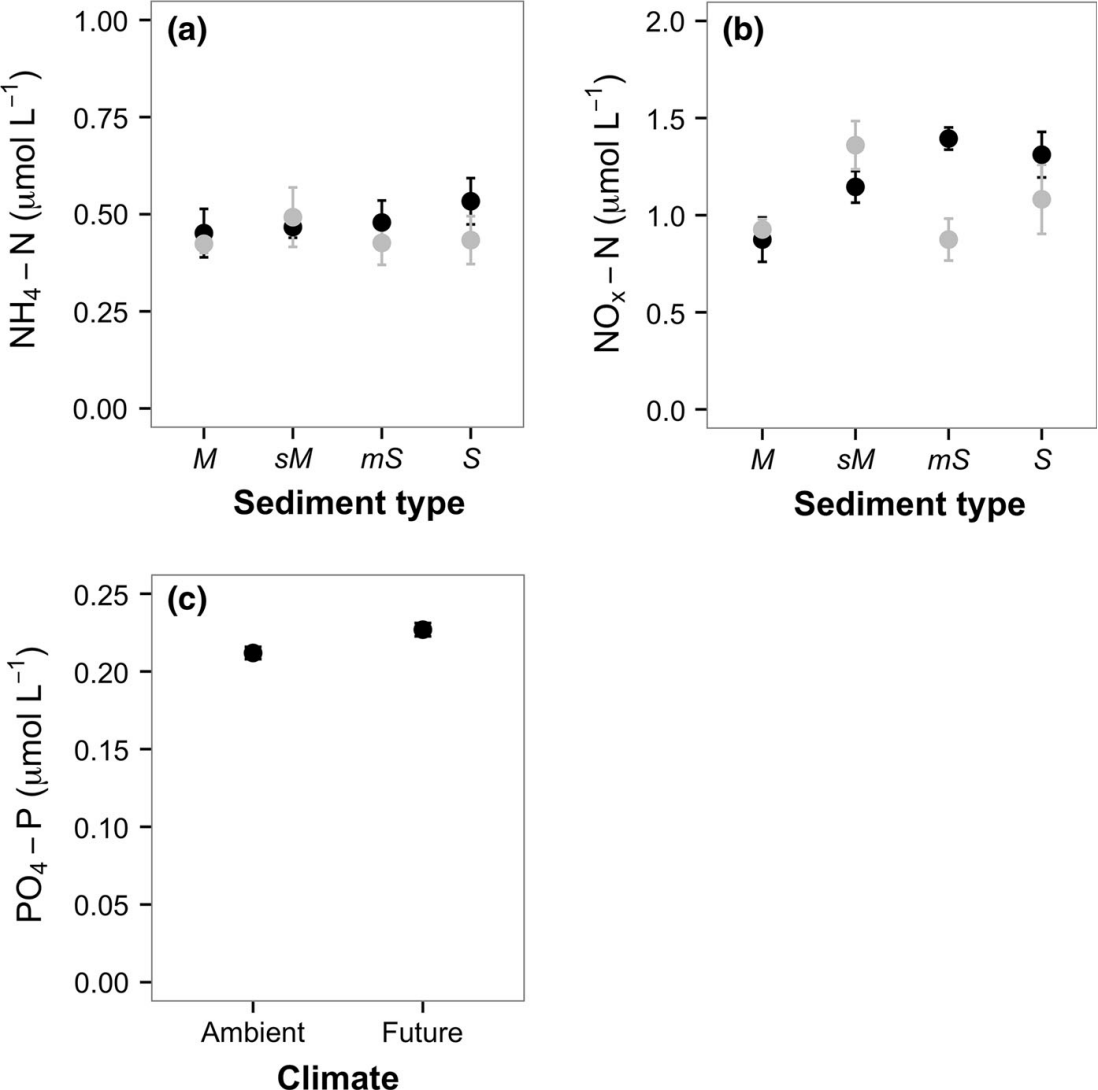
The effects of sediment type and climate regime on mean (±SE, n = 5) nutrient concentrations (μmol L^−1^). We found no effect in **a** [NH_4_-N], interactive effects in **b** [NO_x_-N], and an independent effect of climate regime in **c** [PO_4_-P]. In (**a**, **b**), sediment types are: *M* mud, *sM* sandy mud, *mS* muddy sand, *S* sand; and in (**a**, **c**) climate regimes are indicated by *closed circles*: *black* = ambient (11 °C, 380 ppm [CO_2_]), *grey* = future (15 °C, 1000 ppm [CO_2_]) conditions

The effects of sediment type and climate regime on nutrient concentrations differed between nutrients. [NH_4_-N] was not affected by either sediment type or climate regime (ANOVA intercept only model; Fig. 2a). However, there is a trend of increasing [NH_4_-N] from muddy to sandy sediments and to reduced [NH_4_-N] under future environmental conditions in muddy-sand and sandy sediments (Fig. 2a). [NO_x_-N], however, was influenced by the interactive effects of sediment type × climate regime (ANOVA: F_3,32_ = 4.264, p < 0.05). Overall, under the future climate regime [NO_x_-N] was higher in comparison to the ambient conditions in mud and sandy-mud sediments, but lower in muddy-sand and sandy sediments (Fig. 2b). However, in muddy-sand [NO_x_-N] was lower under future environmental conditions when compared to ambient conditions (coefficient ± SE = −0.572 ± 0.221, t value = −2.583, p < 0.05). [PO_4_-P] changed with climate regime, irrespective of sediment type (ANOVA: F_1,38_ = 6.614, p < 0.05), with higher [PO_4_-P] under future environmental conditions (Fig. 2c).

**Fig. 3:**
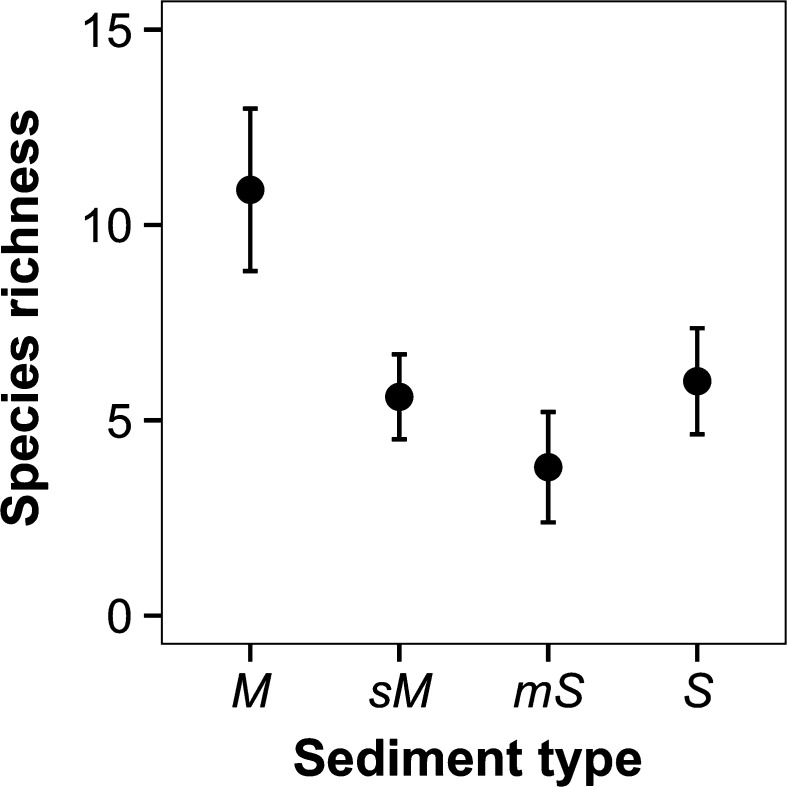
The independent effect of sediment type on mean (±SE, n = 10) macrofaunal species richness. Sediment types are indicated by: *M* mud, *sM* sandy mud, *mS* muddy sand, *S* sand

**Fig. 4:**
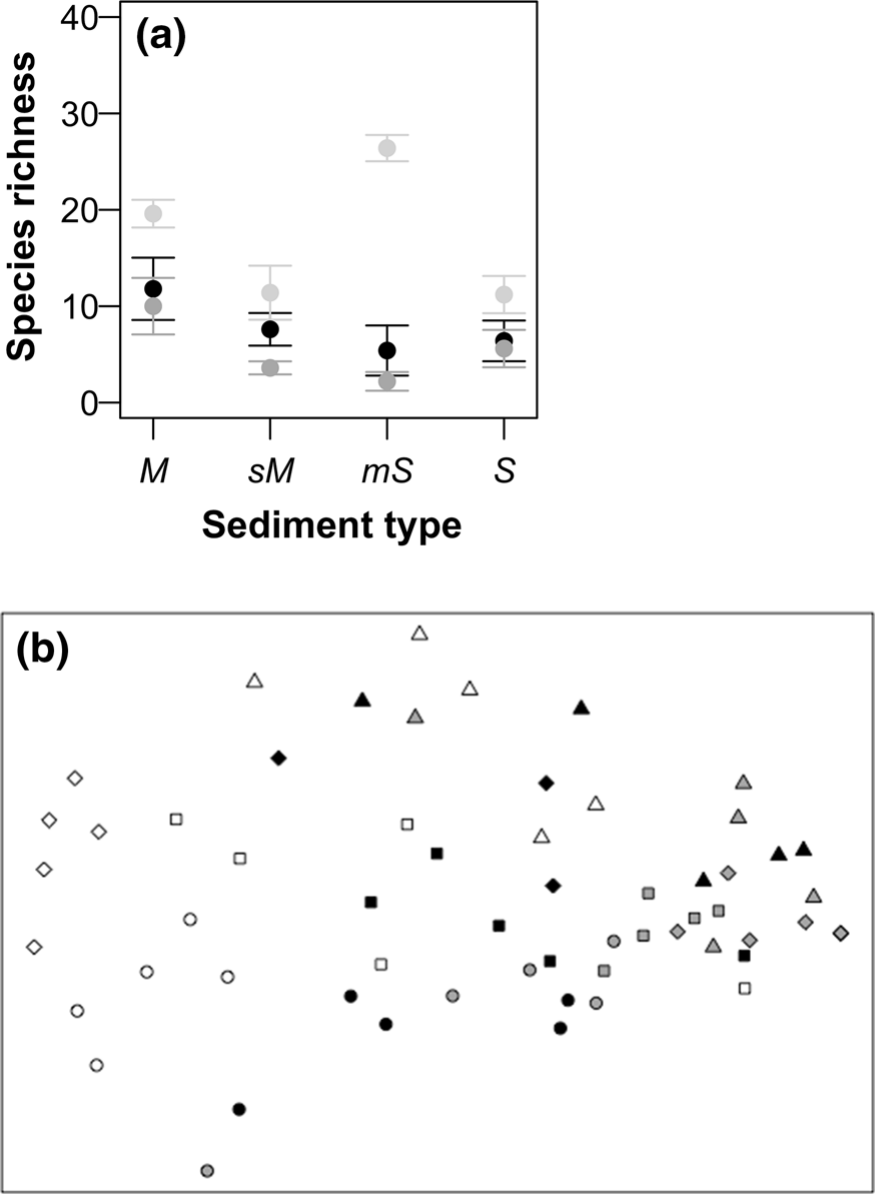
In **a** the interactive effects of sediment type and climate regime on mean (±SE, n = 5) species richness for macrofaunal communities retrieved immediately in the field (*light grey*) or after a 6-month incubation under an ambient (*black*; 11 °C, 380 ppm [CO_2_]) or future (*dark grey*; 15 °C, 1000 ppm [CO_2_]) climate regime. In **b** visualisations represent non-metric two-dimensional multi-dimensional scaling (nMDS) ordinations based on square root transformed zero-adjusted Bray–Curtis dissimilarity matrices of macrofaunal abundance. MDS dimensionality representation stress value = 0.194. Sediment types in **b** are: *M/circle* mud, *sM/square* sandy mud, *mS/diamond* muddy sand, *S/triangle* sand)

Following 6-months incubation in the laboratory, macrofaunal species richness varied between sediment types (ANOVA, F_3_,_35_ = 4.137, p < 0.05), but there was no difference between climate regimes (ANOVA: F_1,35_ = 2.690, p = 0.109). We observed the highest number of species (mean ± SE, n = 10) in muddy sediments (11 ± 2) and the lowest number of species in muddy-sand (4 ± 1) (Fig. 3). In muddy-sand there were three replicates in which there were no macrofauna present (two replicates under ambient and one replicate under future conditions). Total abundance was affected by climate regime (L-ratio = 5.006, df = 1, p < 0.05) (Supplementary material Figure S3a), with a significantly lower density of individuals under future climatic conditions, irrespective of sediment type. Whilst there was no effect on total biomass of sediment type (ANOVA: F_3,35_ = 2.634, p = 0.065) or climate regime (ANOVA: F_1,35_ = 3.580, p = 0.068) (Supplementary material Figure S3b). Comparison of macroinfaunal communities held under laboratory conditions with communities that were immediately recovered after sampling, revealed that there were sediment type and experimental regime dependent differences in species richness (ANOVA, sediment type × climatic scenario, F_6,48_ = 5.065, p < 0.001). Overall mean (±SE) species richness was lowest (2 ± 1) under future climate conditions in muddy-sand and highest (26 ± 1) in muddy-sand that were immediately recovered after sampling (Fig. 4a). Overall species richness decreased from cohesive to non-cohesive sediments across both climate scenarios. We observed differences in macrofaunal community composition when based on abundance (Fig. 4b) between the laboratory and field communities across all sediment types (PERMANOVA, sediment type × laboratory/field regime, F_1,56_ = 1.635, p < 0.05).

**Fig. 5:**
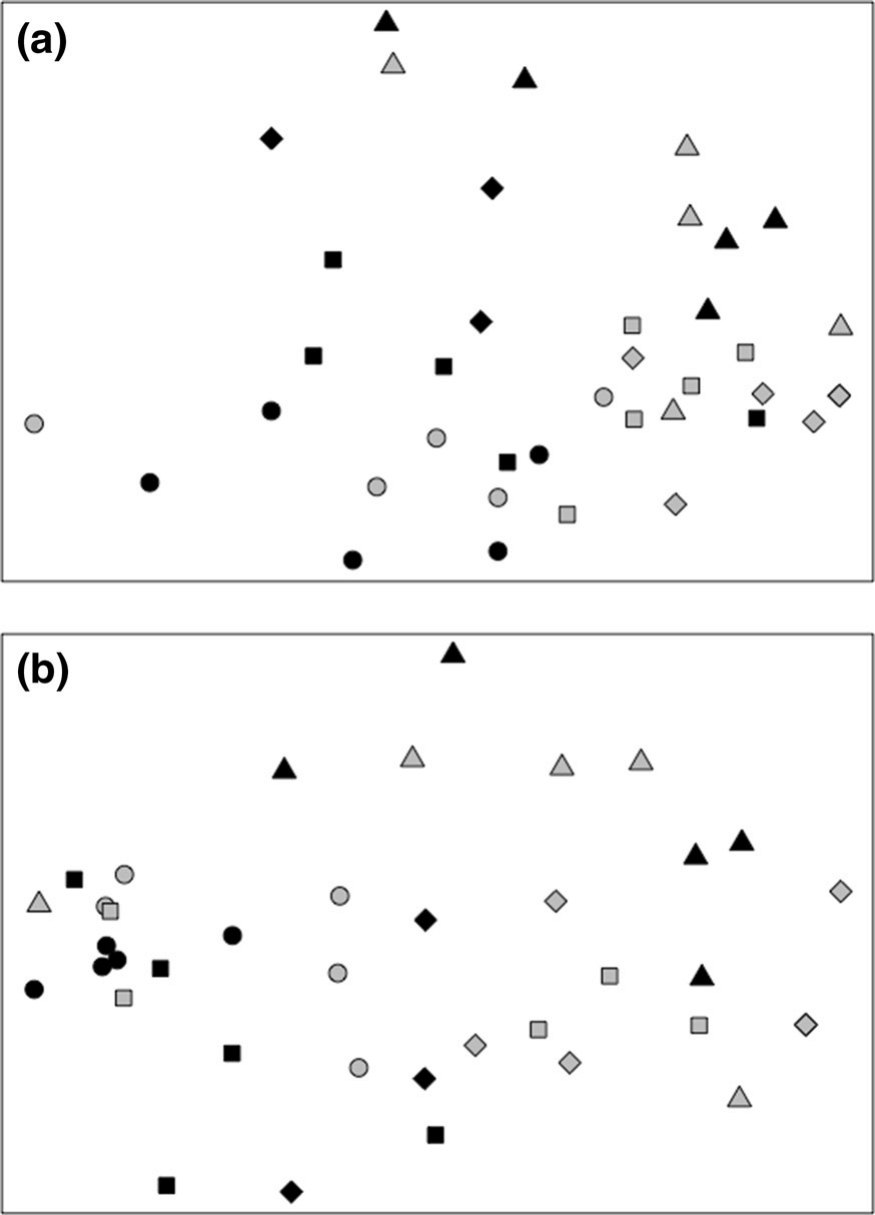
Non-metric two-dimensional multi-dimensional scaling (nMDS) representations of the effects of sediment type and climate regime on macrofaunal community composition. Visualisations are based on square root transformed zero-adjusted Bray–Curtis dissimilarity matrices for **a** macrofaunal abundance and **b** macrofaunal biomass. Climate regimes are indicated by colour (*black* = Ambient: 11 °C, 380 ppm [CO_2_]; *grey* = Future: 15 °C, 1000 ppm [CO_2_]) and sediment types are indicated by *symbol*: *circle* = mud, *square* = sandy mud, *diamond* = muddy sand and *triangle* = sand. MDS dimensionality representation stress values are: **a** 0.185, **b** 0.200 (*top *Abundance,* bottom* Biomass)

We observed changes in macrofaunal community composition when based on sqrt abundance between sediment type (PERMANOVA, F_1,39_ = 7.309, p < 0.05) and between climate regime (PERMANOVA, F_1,39_ = 2.021, p < 0.05) as single terms (Fig. 5a), but not when based on community biomass (PERMANOVA, sediment type: F_1,39_ = 3.867, p = 0.163; climate regime: F_1,39_ = 0.94, p = 0.453; sediment type × climate regime: F_1,39_ = 1.346, p = 0.069, Fig. 5b). SIMPER analysis suggests that differences in species composition based on abundance and between sediment type were related to increased densities of the polychaete *Magelona minuta* and Nematoda at the sandy-mud and muddy-sand in comparison to the sandy site, higher densities of the bivalve *Abra nitida*, the polychaete *Magelona minuta* and Nematoda at the site in comparison to the other sites, as well as high densities of the polychaete *Ophryotrocha* sp. and the clitellatid *Grania* sp. at the sandy site (Supplementary Table S2). Similarly, differences in species composition based on abundance between the ambient and future climate regimes were associated with increased densities of *M. minuta*, Nematoda, *A. nitida* and *Ophryotrocha* sp. (ordered by reducing average contribution to overall dissimilarity between sites) in the ambient treatment (Supplementary Table S2).

**Fig. 6:**
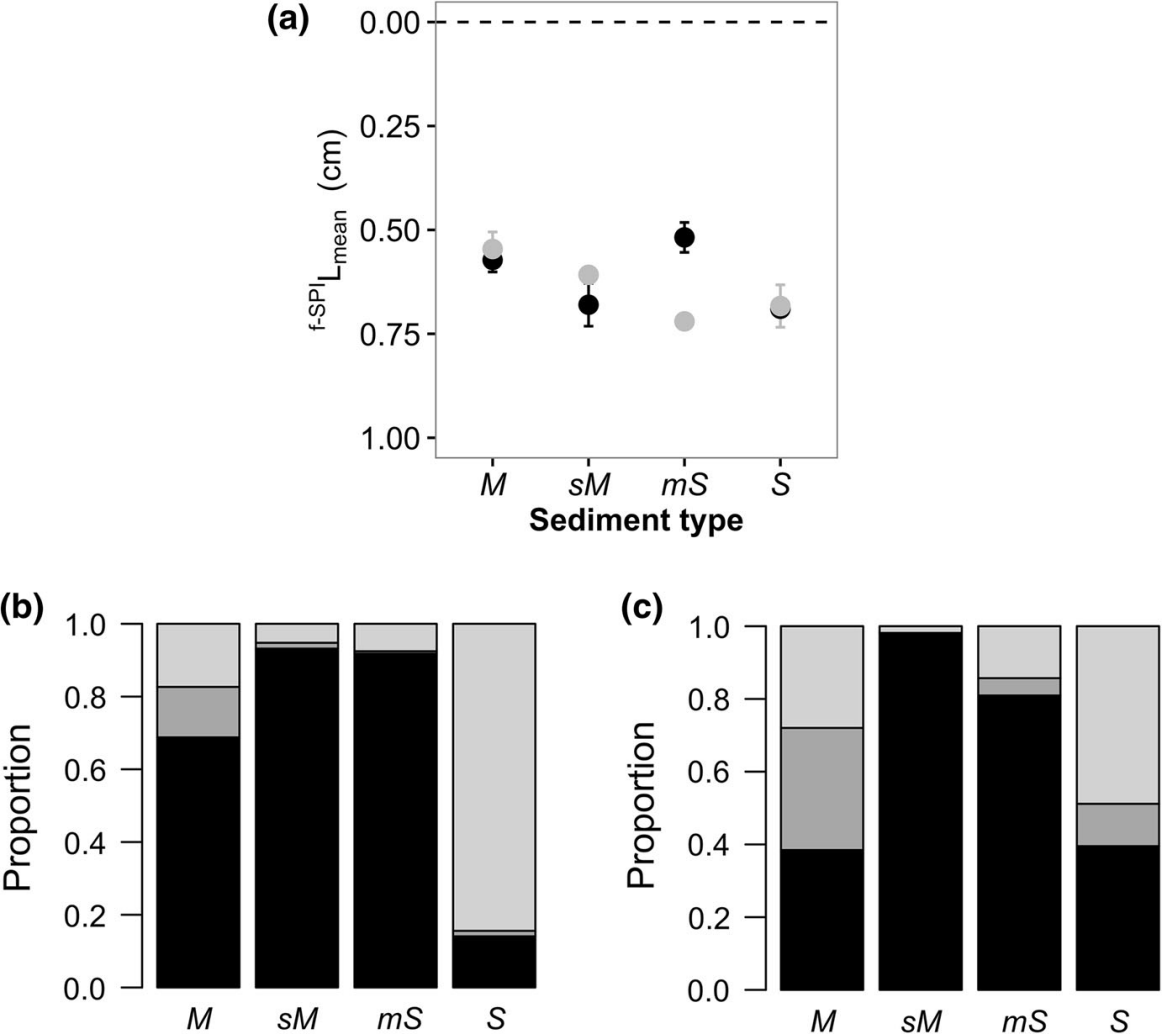
Effects of the interactive effects of sediment type and climate regime on **a** mean particle reworking depth (mean ^f-SPI^L_mean_ ± SE, cm) and **b** the relative distribution of reworking mode groupings pooled across all replicate (n = 5) communities within each sediment type. Climate regimes are indicated by *colour* (*black* Ambient: 11 °C, 380 ppm [CO_2_]; *grey* Future: 15 °C, 1000 ppm [CO_2_]) and sediment types are: *M* mud, *sM* sandy mud, *mS* muddy sand, *S* sand. In panel (**b**) reworking mode groupings are: *black* surficial modifiers, *dark grey* upward/downward conveyors, *light grey* biodiffusers (*left* = ambient,* right *= future)

Faunally mediated particle reworking and burrow ventilation were differentially affected by sediment type and/or climate regime. We find that mean bioturbation depth (^f-SPI^L_mean_) was influenced by the interactive effects of sediment type × climatic scenario (ANOVA: ^f-SPI^L_mean_, F_3,32_ = 5.991, p < 0.01). Overall, ^f-SPI^L_mean_ (mean ± SE, n = 5) increased across the mud:sandy-mud:muddy-sand:sand gradient under both ambient and future climate regimes (Fig. 6a) from 0.57 ± 0.03 cm and 0.55 ± 0.09 cm respectively in muddy sediments to 0.69 ± 0.02 cm and 0.68 ± 0.05 cm respectively in sandy sediments. However, only in muddy-sand was ^f-SPI^L_mean_ shallower in the ambient climate regime (ambient, 0.52 ± 0.04 cm; future, 0.72 ± 0.02 cm). In contrast, maximum bioturbation depth (^f-SPI^L_max_) was not affected, albeit marginally, by sediment type and/or climatic scenario (ANOVA: L_max_, F_3,32_ = 2.799, p = 0.056). In mixed sediments (sandy-mud and muddy-sand) however, future climate conditions had opposing effects on ^f–SPI^L_max_ with a reduction by ~1.32 cm in sandy-mud versus an increase by ~1.22 cm in muddy-sand relative to ambient conditions (Supplementary Figure S4a). Surface boundary roughness (SBR) did not change with sediment type and/or climate regime (ANOVA: SBR, F_7,32_ = 1.374, p = 0.250), although, with the exception of muddy-sand, SBR did show a tendency to be lower in absolute terms under future climate conditions (Supplementary Figure S4b). Bioirrigation activity (ΔBr^−^) was also not affected by sediment type and/or climate regime (ANOVA sediment type × climatic scenario, F_7,32_ = 2.016, p = 0.085). Patterns suggest however, that bioirrigation increased across the mud:sandy-mud:muddy-sand:sand gradient under future climate conditions, although differences were only significant in sandy sediments (Supplementary Fig. S4c).

Separating species into functional groups based on reworking mode highlighted considerable differences in the relative distribution of functional groups across sediment types and between climate regimes (Figs. 6, S6). Under the future climate regime in muddy sediments, we observed a reduction in surficial modifiers and an increase in conveyors and biodiffusers, whilst in sandy sediments we observed an overall reduction in biodiffusers and an increase in surficial modifiers relative to the ambient climate regime.

## Discussion

Our motivation was to contribute to the understanding of the controlling abiotic and biotic mechanisms that support shelf sea sediment carbon and nutrients, and to provide insight as to how primary habitats may respond to anticipated climatic forcing (IPCC 2014). Improved projections of the ecological consequences of warming and ocean acidification requires better understanding of longer-term processes (Form and Riebesell 2012; Godbold and Solan 2013; Tatters et al. 2013) that moderate the susceptibility of species and ecosystems to changing environmental conditions. The present study was not designed to investigate the adaptation potential of shelf-sea communities or individual species, which would involve a shift in genotype or phenotype over several generations (Ghalambor et al. 2007). Rather, we investigate organism/community and biogeochemical responses to an extended period (6 months) of exposure to warmer temperature and reduced pH in order to incorporate acclimation processes.

In the broadest terms, our results might suggest that bulk inventories of organic carbon, nitrogen and phosphate associated with shelf sea sediment communities of the Celtic Sea are, irrespective of sediment type, largely unaffected by a simultaneous increase in temperature and decrease in pH. We could also conclude that there is a consistent, albeit statistically insignificant, trend of increasing nutrient concentrations with increasing sediment particle size that challenges the commonly held view that the focus of biogeochemical activity tracks sediment organic carbon content (Hedges and Keil 1995). Indeed, many of the effects we have observed were driven by the response of muddy-sands, indicating that non-cohesive sediments play a major role in the turnover of particulate organic matter (Rocha 2008). These observations are not trivial, as many studies seeking to understand cycles of nutrients have focused on establishing differences between the biogeochemical performance of cohesive versus non-cohesive sediments, without considering intermediate or mixed sediments that extend over significant portions of the shelf sea (Thompson et al. 2017). We contend, however, that the above conclusions are misguided because they ignore substantive changes in the diversity, composition and moderating affect of the biological community. For instance, we observed a decline in [NO_x_-N] under future conditions relative to ambient conditions in muddy sands, suggesting that an adjustment to N-cycling processes may have occurred, including dissimilatory nitrate reduction and denitrification, or via a reduction in nitrification (Kitidis et al. 2011, 2017). Species richness and total abundance was lowest in muddy-sands under future environmental conditions, indicating that a reduction in bioturbating fauna may have negatively affected nitrification. Indeed, enhanced microbial activity and nitrification rates in the burrow walls of bioturbating fauna can be disrupted by low surface water pH conditions (Laverock et al. 2011, 2013), although the extent to which pH associated reductions in substrate availability take place during ocean acidification cannot be discounted (Suzuki et al. 1974). It is also important to consider our findings within the context of the changes in carbonate chemistry observed in our experimental system. In particular, we observed an elevated level of alkalinity in our future climate treatments, which may be a result an increase in sediment carbonate dissolution due to ocean acidification (Gattuso and Hansson 2011). Concurrent with findings elsewhere (Laverock et al. 2013), increased carbonate dissolution within sediment porewaters is likely to have buffered the reduction in pH and, therefore, microbial mediation of nutrients (Tait et al. 2013). This indicates that the level of acidification, and the associated faunal responses, we observed are conservative relative to what may happen if pH buffering had not occurred.

Communities in sandy-mud and muddy-sand were dominated by surficial modifiers and the functional composition of the communities did not considerably alter between ambient and future climatic conditions. In contrast, mud communities were dominated by surficial modifiers and sand communities were dominated by biodiffusers under ambient conditions, but underwent considerable change in functional composition (mud: increase in conveyors, decrease in surficial modifiers; sand, reduction in biodiffusers, increase in surficial modifiers and conveyors) under future climatic conditions (Widdicombe et al. 2009). It follows that the extent to which individual species or assemblages control biogeochemical processes will vary with environmental context (Godbold and Solan 2009) and depend upon the relevance and relative importance of individual functional traits to specific microbial processes and/or nutrient pathways (Murray et al. 2014; Hale et al. 2014; Botto et al. 2005; Laverock et al. 2010). We conclude, therefore, that the effects of climatic forcing on biogeochemical condition are predominantly expressed through functionally important changes in microbial and macrofaunal community structure, and their interactions (Gilbertson et al. 2012), rather than via changes in carbonate chemistry and metabolism associated with an altered environment (Drupp et al. 2016; Kim 2016).

Whilst many studies lack the necessary interdisciplinary focus to distinguish the relative role of different components of a natural system, we concur with findings elsewhere (e.g. Eklöf et al. 2015) that the susceptibility of individual species to multiple stressors and the biogeochemical consequences of species loss are relatively predictable (Busch and McElhany 2016; Queirós et al. 2013; Solan et al. 2004b; Thomsen et al. in press). A priority challenge must be elucidating which components of natural systems are important in determining whole system net responses to forcing, when such mechanisms may be at risk, and how multiple stressors interact with one another to alter the mechanistic balance. The limited quantitative understanding of how species behavior and activity couple with the processes that underpin biogeochemical condition contrasts with the potential significance of shelf sea sediments for the global carbon and nutrient cycles. It is important to consider that the processes involved in determining biogeochemical performance are highly dynamic and respond to a variety of interacting factors that are not generally incorporated in experimental designs, including the role of environmental context (season, Godbold and Solan 2013; Braeckman et al. 2014; presence of additional stressors, Hale et al. 2017; Sciberras et al. 2017). There are examples emerging in the literature of both additive and interactive (Bulling et al. 2010; Kroeker et al. 2013) effects of multiple climatic stressors on biogeochemical performance in the presence of bioturbating fauna, but the number of experiments is low and the focus tends to be on ocean acidification (e.g. Kitidis et al. 2011; Gazeau et al. 2014). We note that, if the reduction in species richness shows a preferential loss of species with smaller body size, it follows that the potential to detect significant effects of ocean acidification and temperature in naturally assembled communities with the full complement of species is reduced because larger species tend to survive and disproportionately contribute to bioturbation activity (Solan et al. 2004b; Thomsen et al. 2017). However, we observed that vulnerability to changes in environmental context can span the full range of body size, and that any associated changes in biogeochemistry may be more dependent upon the functional role of the species and whether or not species behaviour changes with environmental (Godbold and Solan 2013) or biotic (Maire et al. 2010) context. Indeed, our data indicates that where the relative proportion of functional groups deviated most between climate regimes (here, muddy-sand) communities exhibited low functional redundancy and the biogeochemical consequences were greatest. Whilst we have shown that biogeochemical processes are closely controlled by faunal traits and that the most biogeochemically vulnerable communities exhibit low functional redundancy, it is clear that an improved description of both functional effect and functional response traits at the community level is needed if we are to appropriately represent organism-sediment relations in predictive models (Yool et al. 2013).

## Electronic supplementary material

Below is the link to the electronic supplementary material.

Supplementary material 1 (PDF 1085 kb)
