# Supplementary material for: Vulnerability of macronutrients to the concurrent effects of enhanced temperature and atmospheric pCO2 in representative shelf sea sediment habitats
Source: Biogeochemistry. 2017 Jun 9;135(1):89–102. doi: 10.1007/s10533-017-0340-y (PMC6961501; doi:10.1007/s10533-017-0340-y)
Supplement: Supplementary file 1 — Supplementary material 1 (PDF 1085 kb) [file 10533_2017_340_MOESM1_ESM.pdf]

SUPPLEMENTARY MATERIAL

Vulnerability of macronutrients to the concurrent effects of enhanced temperature and atmospheric pCO_2 in representative shelf sea sediment habitats

Jasmin A. Godbold[1,2], Rachel Hale[1], Christina L. Wood[1], Martin Solan[1]

[1]Ocean and Earth Science, University of Southampton, National Oceanography Centre Southampton, European Way, Southampton, SO14 3ZH, UK.

[2]Biological Sciences, University of Southampton, Highfield Campus, Southampton, SO17 1BJ, UK.

Corresponding Author: Jasmin A. Godbold

Email: j.a.godbold@soton.ac.uk

Phone: +44 (0) 2380 593639

Figure S1 (next 4 pages): Summary of the seawater carbonate conditions in the experimental aquaria during the 6-month incubation period of sediment cores containing intact macrofaunal communities from the a) mud, b) sandy-mud, c) muddy-sand and d) sandy site in the Celtic Sea. • ambient conditions (11°C and [CO_2] 380ppm), • = future conditions (15°C and [CO_2] 1000ppm). Temperature (°C), Salinity, pH_{NBS} and total alkalinity (A_T, µmol kgSW-1) were measured directly from each mesocosm (3 replicates month^{-1}, 5 replicates for the final measurements after 6 months) and were used to calculate dissolved organic carbon (DIC, µmol kgSW-1), pCO_2SW (µAtm), saturation states for calcite (ΩCalcite) and aragonite (ΩAragonite), bicarbonate (HCO_3, µmol kgSW^{-1}) and carbonate (CO_3, µmol kgSW-1) using *CO2calc* (Robbins, L.L., Hansen, M.E., Kleypas, J.A. & Meylan, S.C. 2010 CO2calc - A user-friendly seawater carbon calculator for Windows, Max OS X, and iOS (iPhone). U.S. Geological Survey Open-File Report 2010–1280, 17 pp.).

Figure S1(a) Muddy sediment

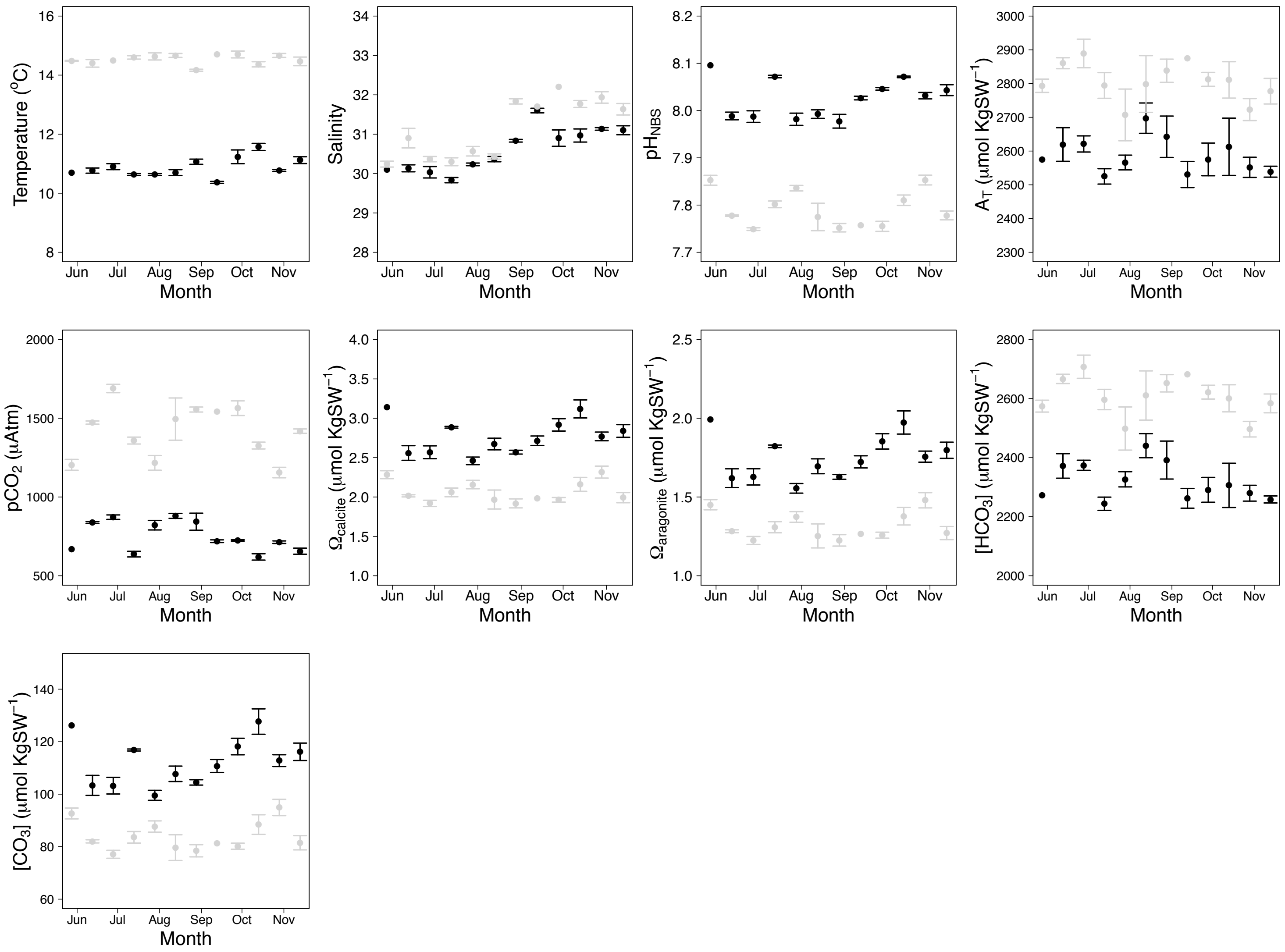

Figure S1(b): Sandy-mud

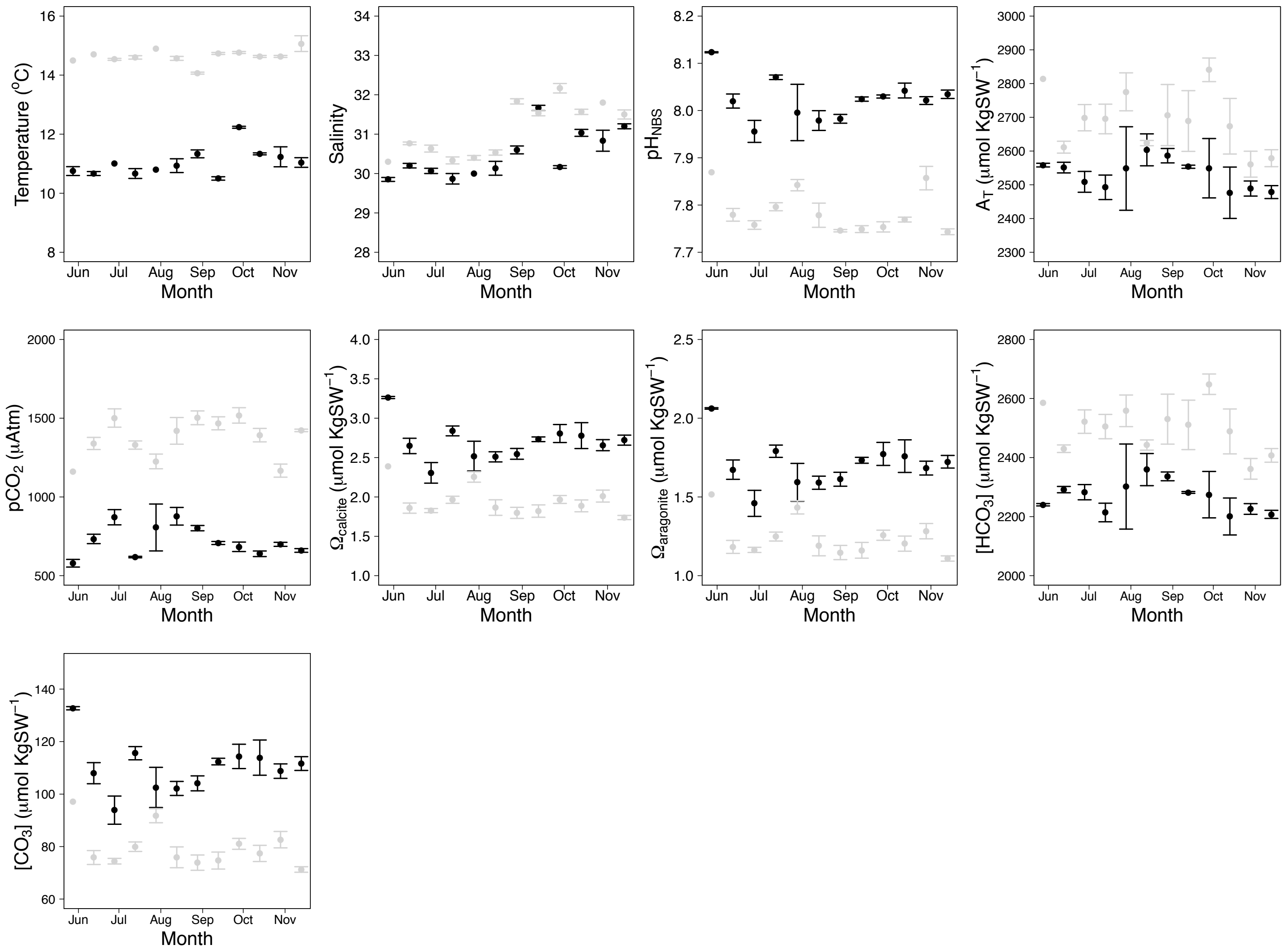

Figure S1 (c): Muddy-sand

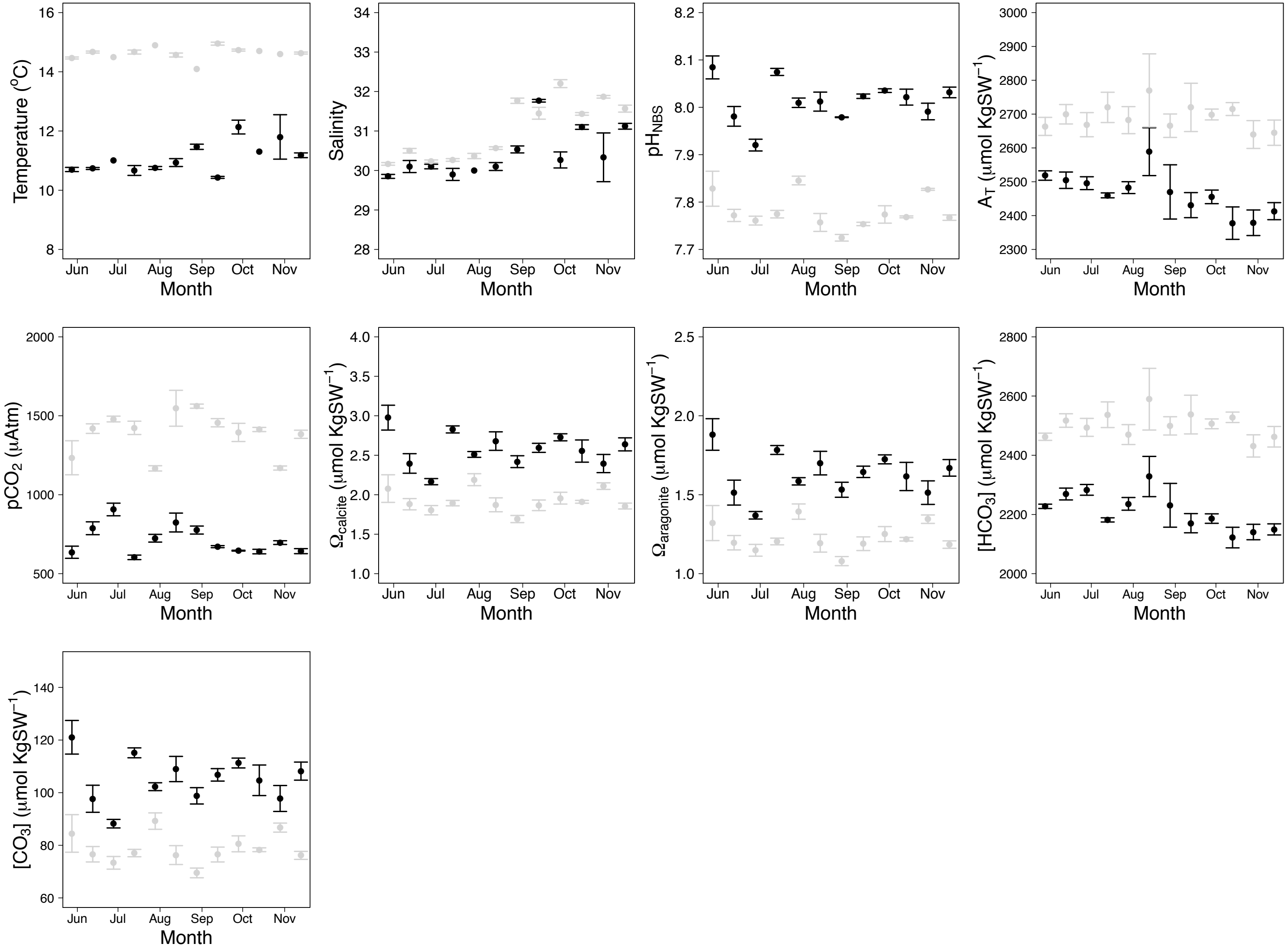

Figure S1 (d): Sandy sediment

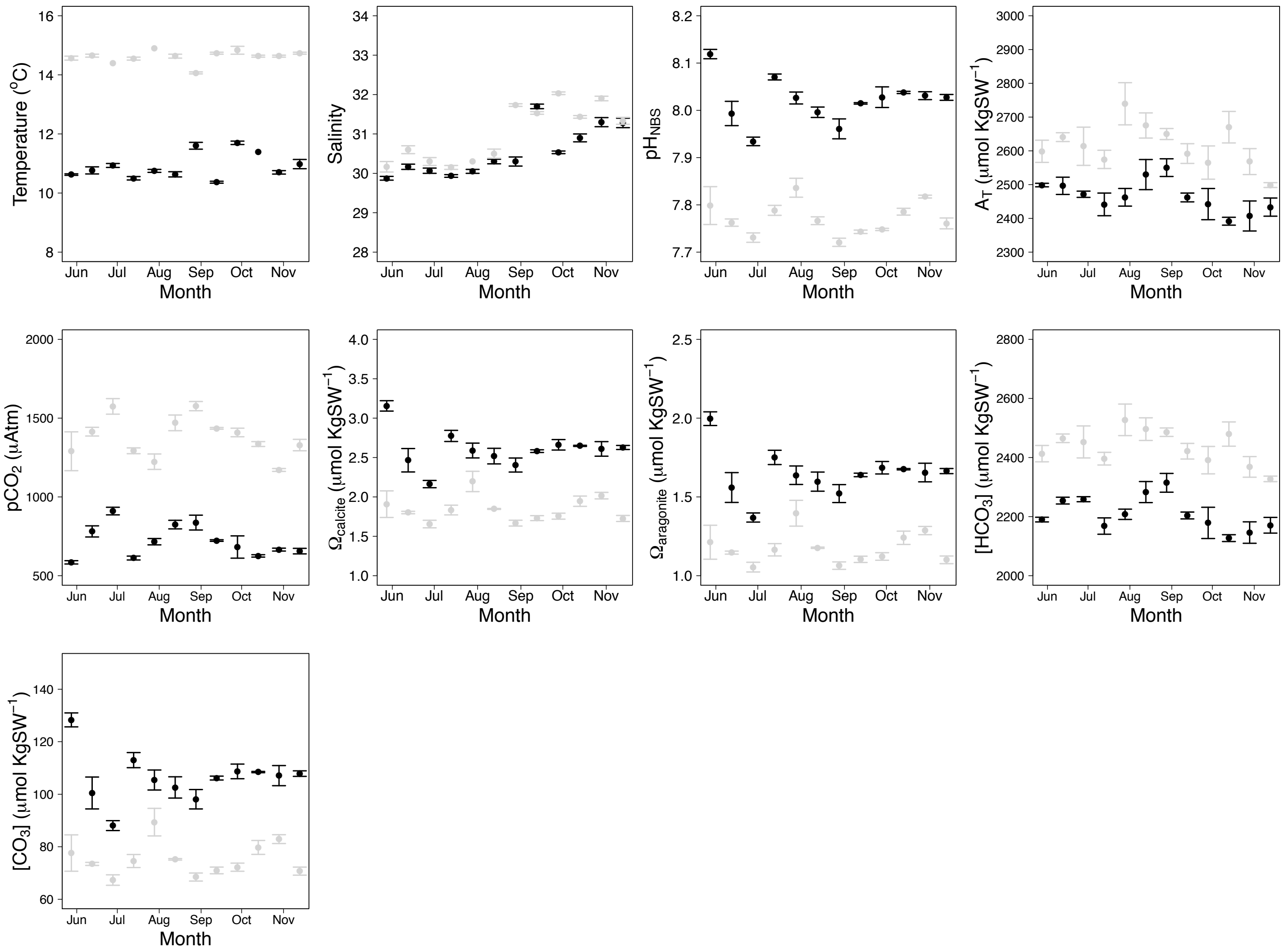

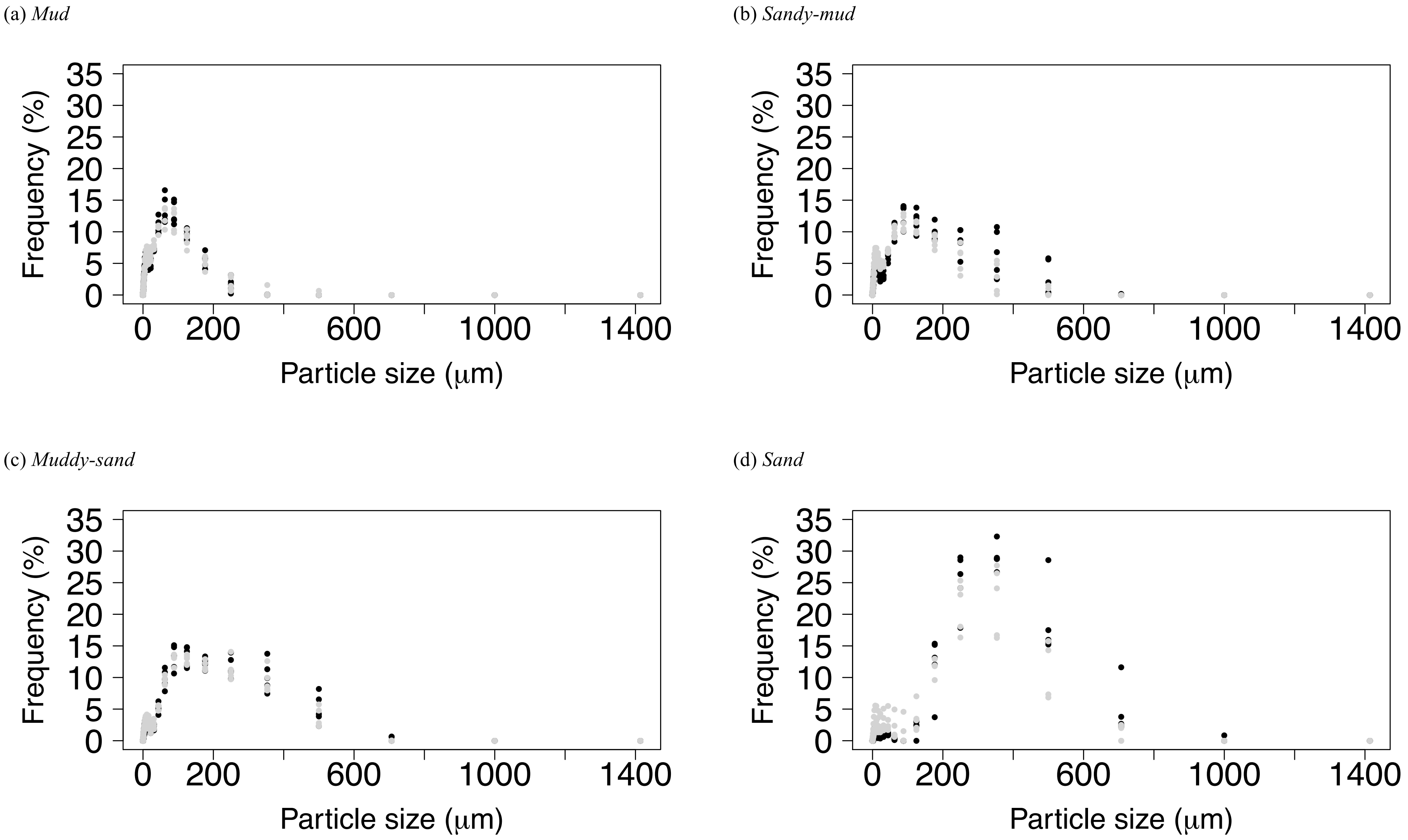

Figure S2: Sediment grain size distribution in the experimental aquaria collected from the a) mud, b) sandy-mud, c) muddy-sand and d) sandy site in the Celtic Sea. Samples were taken from each aquarium after 6 months exposure to ambient (•, 11°C and [CO_2] 380ppm) or future (•, 15°C and [CO_2] 1000ppm) environmental conditions.

Table S1: Summary of sediment characteristics in the experimental aquaria collected from the a) mud, b) sandy-mud, c) muddy-sand and d) sandy site in the Celtic Sea. Samples were taken from each aquarium after 6 months exposure to ambient (11°C and [CO_2] 380ppm) or future (15°C and [CO_2] 1000ppm) environmental conditions.

Site	Environmental condition	Median (d0.5, µm)	Mean	Kurtosis (µm)	Skewness (µm)
**Mud**	Ambient	50.52 ± 7.82	4.77 ± 0.16	0.852 ± 0.043	-0.346 ± 0.080
	Future	46.93 ± 10.36	4.80 ± 0.23	0.882 ± 0.069	-0.286 ± 0.079
**Sandy-mud**	Ambient	96.34 ± 15.96	3.98 ± 0.29	0.900 ± 0.099	-0.382 ± 0.041
	Future	60.58 ± 19.32	4.50 ± 0.39	0.826 ± 0.066	-0.274 ± 0.078
**Muddy-sand**	Ambient	137.12 ± 24.17	3.20 ± 0.42	1.272 ± 0.120	-0.326 ± 0.088
	Future	123.23 ± 19.85	3.52 ± 0.33	1.178 ± 0.139	-0.384 ± 0.018
**Sand**	Ambient	364.94 ± 51.76	1.61 ± 0.37	2.070 ± 0.305	-0.370 ± 0.016
	Future	270.93 ± 75.84	2.59 ± 0.72	1.770 ± 0.935	-0.572 ± 0.069

Figure S3: Impacts of climate regime and sediment type on total community a) abundance (individuals m^{-2}) and b) biomass (g m^{-2}) of macroinfauna in the Celtic Sea. Sediment types are: M = mud, sM = sandy mud, mS = muddy sand, S = sand; the climate regimes are: black circles = Ambient (11°C, 380ppm [CO_2]), grey circles = Future (15°C, 1000ppm [CO_2]) conditions. In a) only climate regime had a significant impact (<0.05), whilst in b) the effects of both sediment type and climate regime were marginal (p = 0.065 and p = 0.67 respectively).

a) b)

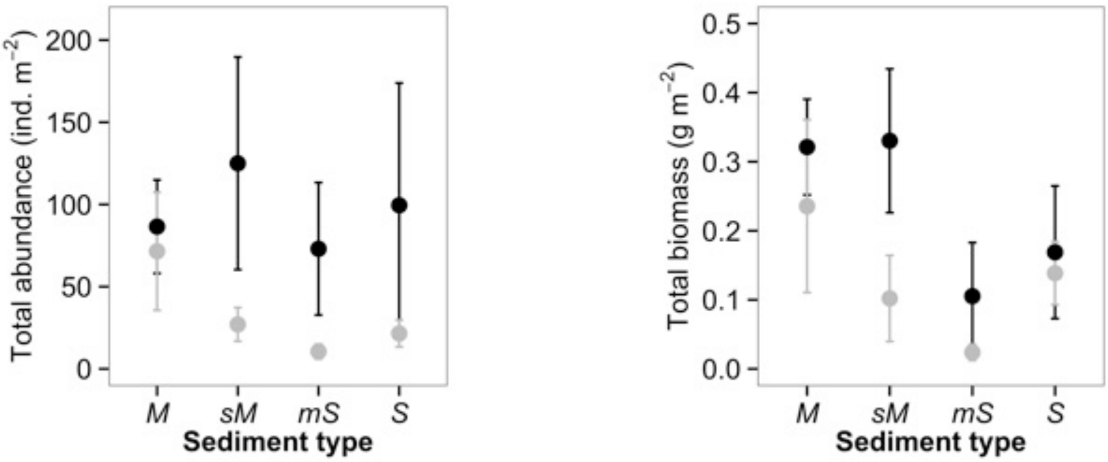

Table S2: Simper Analysis of the relative contribution of individual species driving community effects based on differences in species sqrt abundance between sediment type and climate regime. Sediment types are: M = mud, sM = sandy mud, mS = muddy sand, S = sand; the climate regimes are: Ambient (11°C, 380ppm [CO_2]), Future (15°C, 1000ppm [CO_2]) conditions. Mean contr. = average proportional contribution of species to the overall dissimilarity between treatments, sd = standard deviation of the mean contribution, ratio = mean contribution to sd ratio, Ambient abundance = average abundance per species core^{-1} under ambient conditions, Future abundance = average abundance per species core^{-1} under future conditions, Cum. Sum = ordered cumulative contribution to overall dissimilarity between treatments.

Comparison between Ambient and Future climate regimes

	Mean contribution	sd	ratio	Ambient abundance	Future abundance	Cum. sum
Magelona minuta	0.10126	0.09813	1.03190	2.26	1.09	0.140
Nematoda	0.08278	0.06882	1.20280	1.98	0.97	0.254
Abra nitida	0.04914	0.06122	0.80280	0.81	0.47	0.322
Ophryotrocha sp	0.04779	0.07727	0.61840	0.68	0.64	0.388
Grania	0.03085	0.05712	0.54010	0.49	0.07	0.431
Abyssoninoe hibernica	0.02254	0.03076	0.73270	0.44	0.25	0.462
Echinocyamus pusillus	0.01933	0.03348	0.57720	0.27	0.19	0.488
Corbula gibba	0.01855	0.04506	0.41160	0.05	0.27	0.514
Nucula sulcata	0.01841	0.03080	0.59780	0.15	0.34	0.539
Diplocirrus glaucus	0.01755	0.02371	0.74020	0.39	0.26	0.564
Paraonidae indet	0.01529	0.03125	0.48940	0.24	0.10	0.585
Nemertea	0.01314	0.02209	0.59490	0.27	0.12	0.603
Paradoneis lyra	0.01310	0.02750	0.47620	0.26	0.05	0.621
Tubificoides amplivasatus	0.01296	0.02790	0.46430	0.26	0.05	0.639
Aspidosiphon mulleri	0.01169	0.02517	0.46440	0.12	0.15	0.655
Nucula tenuis	0.01107	0.02367	0.46780	0.23	0.10	0.670
Scoloplos armiger	0.01063	0.02635	0.40340	0.12	0.17	0.685
Harpinia antennaria	0.01042	0.03076	0.33890	0.07	0.10	0.699

Exogone dispar	0.00855	0.01916	0.44650	0.15	0.05	0.711
Urothoe elegans	0.00797	0.01892	0.42090	0.15	0.05	0.722
Edwardsiidae	0.00791	0.01989	0.39760	0.15	0.05	0.733
Exogone hebes	0.00789	0.01748	0.45140	0.17	0.07	0.744
Glycera lapidum	0.00780	0.03606	0.21640	0.10	0.00	0.755
Aricidea cerrutii	0.00775	0.01920	0.40370	0.17	0.00	0.765
Magelona indet	0.00716	0.02253	0.31770	0.20	0.00	0.775
Nephtys hystricis	0.00644	0.01844	0.34920	0.10	0.05	0.784
Aricidea suecica	0.00606	0.01509	0.40140	0.21	0.00	0.792
Levinsenia gracilis	0.00553	0.01522	0.36340	0.05	0.10	0.800
Aricidea indet	0.00533	0.01614	0.33040	0.05	0.10	0.807
Aricidea catherinae	0.00503	0.01650	0.30460	0.09	0.05	0.814
Amphictene auricoma	0.00493	0.01525	0.32290	0.10	0.00	0.821
Glycera fallax	0.00474	0.02236	0.21210	0.05	0.00	0.828
Syllis amica	0.00439	0.02382	0.18420	0.00	0.05	0.834
Syllis cornuta	0.00429	0.01926	0.22260	0.09	0.00	0.840
Pholoe baltica	0.00417	0.01470	0.28390	0.10	0.00	0.846
Iphinoe serrata	0.00409	0.01986	0.20600	0.00	0.07	0.851
Ophiodromus flexuosus	0.00382	0.01231	0.31080	0.05	0.05	0.856
Praxillella affinis	0.00369	0.01171	0.31540	0.07	0.12	0.862
Praxiella sp	0.00369	0.01171	0.31540	0.07	0.12	0.867
Prionospio minuspio	0.00369	0.01171	0.31540	0.07	0.12	0.872
Prionospio sp	0.00369	0.01171	0.31540	0.07	0.12	0.877
Pseudomystides limbata	0.00369	0.01171	0.31540	0.07	0.12	0.882
Pseudomystides sp	0.00369	0.01171	0.31540	0.07	0.12	0.887
Pseudonotomastus southerni	0.00369	0.01171	0.31540	0.07	0.12	0.892
Pseudopolydora pulchra	0.00369	0.01171	0.31540	0.07	0.12	0.897
Scalibregma inflatum	0.00369	0.01171	0.31540	0.07	0.12	0.902
Scolelepis foliosus	0.00369	0.01171	0.31540	0.07	0.12	0.907
Glyphohesione klatti	0.00337	0.01045	0.32240	0.12	0.00	0.912

Ophiuroidea juv	0.00331	0.01465	0.22600	0.11	0.00	0.917
Mediomastus fragilis	0.00325	0.01064	0.30530	0.10	0.00	0.921
Amphiura chiajei	0.00303	0.01374	0.22040	0.05	0.00	0.925
Golfingia elongata	0.00303	0.01374	0.22040	0.05	0.00	0.929
Aonides paucibranchiata	0.00303	0.00975	0.31050	0.05	0.05	0.934
Golfingia sp	0.00298	0.01455	0.20480	0.00	0.05	0.938
Prionospio fallax	0.00295	0.01336	0.22070	0.05	0.00	0.942
Notomastus sp	0.00289	0.01404	0.20600	0.00	0.05	0.946
Thyasira flexuosa	0.00266	0.01275	0.20890	0.00	0.05	0.950
Prionospio multibranchiata	0.00258	0.01230	0.21000	0.00	0.05	0.953
Dosinia exoleta	0.00248	0.01112	0.22260	0.05	0.00	0.957
Apistobranchus tullbergi	0.00219	0.00982	0.22360	0.05	0.00	0.960
Retusa truncatula	0.00219	0.00982	0.22360	0.05	0.00	0.963
Leucon nasica	0.00219	0.00982	0.22360	0.05	0.00	0.966
Ancistrosyllis groenlandica	0.00195	0.00869	0.22450	0.05	0.00	0.968
Laubieriellus salzi	0.00195	0.00869	0.22450	0.05	0.00	0.971
Goneplax rhomboides	0.00195	0.00869	0.22450	0.05	0.00	0.974
Nephtys incisa	0.00178	0.00815	0.21880	0.00	0.05	0.976
Caulleriella zetlandica	0.00176	0.00805	0.21900	0.00	0.05	0.979
Peresiella clymenoides	0.00176	0.00805	0.21900	0.00	0.05	0.981
Nucula nucleus	0.00166	0.00738	0.22540	0.05	0.00	0.983
Cirrophorus furcatus	0.00148	0.00655	0.22600	0.05	0.00	0.985
Glycinde nordmanii	0.00148	0.00655	0.22600	0.05	0.00	0.987
Magelona filiformis	0.00148	0.00655	0.22600	0.05	0.00	0.989
Syllis sp	0.00148	0.00655	0.22600	0.05	0.00	0.991
Harpinia sp	0.00148	0.00655	0.22600	0.05	0.00	0.994
Abra prismatica	0.00137	0.00606	0.22630	0.05	0.00	0.995
Amphiura filiformis	0.00137	0.00606	0.22630	0.05	0.00	0.997
Prionospio caspersi	0.00105	0.00463	0.22720	0.05	0.00	0.999
Nepthys sp	0.00094	0.00414	0.22580	0.00	0.05	1.000

Comparison between Mud and Sand habitats

	Mean contribution	sd	ratio	Mud abundance	Sand abundance	Cum. sum
Magelona minuta	0.07666	0.03870	1.98090	1.92	0.00	0.094
Abra nitida	0.07147	0.05979	1.19530	1.64	0.24	0.181
Nematoda	0.06210	0.03709	1.67440	1.93	0.64	0.257
Ophryotrocha sp	0.05872	0.07863	0.74680	0.51	1.82	0.329
Abyssoninoe hibernica	0.04770	0.02638	1.80810	1.19	0.00	0.388
Grania	0.03918	0.04279	0.91560	0.14	0.97	0.435
Echinocyamus pusillus	0.02952	0.02737	1.07880	0.00	0.77	0.472
Diplocirrus glaucus	0.02420	0.02515	0.96220	0.68	0.27	0.501
Nucula tenuis	0.02395	0.02919	0.82060	0.67	0.00	0.531
Nucula sulcata	0.02177	0.03154	0.69020	0.50	0.00	0.557
Aspidosiphon mulleri	0.01765	0.02410	0.73260	0.00	0.44	0.579
Nemertea	0.01494	0.02098	0.71180	0.48	0.00	0.597
Tubificoides amplivasatus	0.01472	0.02346	0.62780	0.14	0.30	0.615
Scoloplos armiger	0.01225	0.02167	0.56530	0.39	0.10	0.630
Corbula gibba	0.01197	0.02691	0.44490	0.20	0.10	0.645
Paraonidae indet	0.01130	0.01892	0.59740	0.00	0.38	0.659
Exogone hebes	0.01111	0.01810	0.61400	0.00	0.38	0.672
Edwardsiidae	0.01082	0.02105	0.51380	0.20	0.10	0.685
Aricidea cerrutii	0.01012	0.02118	0.47780	0.24	0.00	0.698
Levinsenia gracilis	0.00998	0.02138	0.46650	0.20	0.00	0.710
Glycera lapidum	0.00973	0.03135	0.31040	0.00	0.20	0.722
Aricidea indet	0.00886	0.01708	0.51900	0.20	0.10	0.733
Magelona indet	0.00851	0.02613	0.32580	0.26	0.00	0.743
Ophiodromus flexuosus	0.00822	0.01701	0.48320	0.20	0.00	0.753

Pholoe baltica	0.00793	0.01886	0.42080	0.20	0.00	0.763
Praxillella affinis	0.00784	0.01655	0.47360	0.39	0.00	0.773
Praxiella sp	0.00784	0.01655	0.47360	0.39	0.00	0.782
Prionospio minuspio	0.00784	0.01655	0.47360	0.39	0.00	0.792
Prionospio sp	0.00784	0.01655	0.47360	0.39	0.00	0.801
Pseudomystides limbata	0.00784	0.01655	0.47360	0.39	0.00	0.811
Pseudomystides sp	0.00784	0.01655	0.47360	0.39	0.00	0.821
Pseudonotomastus southerni	0.00784	0.01655	0.47360	0.39	0.00	0.830
Pseudopolydora pulchra	0.00784	0.01655	0.47360	0.39	0.00	0.840
Scalibregma inflatum	0.00784	0.01655	0.47360	0.39	0.00	0.849
Scolelepis foliosus	0.00784	0.01655	0.47360	0.39	0.00	0.859
Paradoneis lyra	0.00753	0.01784	0.42200	0.00	0.20	0.868
Mediomastus fragilis	0.00627	0.01383	0.45310	0.20	0.00	0.876
Syllis cornuta	0.00616	0.01943	0.31720	0.00	0.17	0.883
Thyasira flexuosa	0.00616	0.01941	0.31740	0.10	0.00	0.891
Syllis amica	0.00603	0.01977	0.30480	0.00	0.10	0.898
Harpinia antennaria	0.00603	0.01977	0.30480	0.00	0.10	0.906
Prionospio multibranchiata	0.00596	0.01875	0.31790	0.10	0.00	0.913
Prionospio fallax	0.00557	0.01746	0.31910	0.10	0.00	0.920
Aonides paucibranchiata	0.00534	0.01131	0.47220	0.00	0.20	0.926
Golfingia sp	0.00472	0.01518	0.31110	0.00	0.10	0.932
Aricidea suecica	0.00461	0.01402	0.32870	0.22	0.00	0.938
Apistobranchus tullbergi	0.00421	0.01302	0.32300	0.10	0.00	0.943
Retusa truncatula	0.00421	0.01302	0.32300	0.10	0.00	0.948
Leucon nasica	0.00421	0.01302	0.32300	0.10	0.00	0.953
Nephtys incisa	0.00401	0.01241	0.32350	0.10	0.00	0.958
Aricidea catherinae	0.00388	0.01196	0.32410	0.00	0.17	0.963
Amphictene auricoma	0.00356	0.01122	0.31720	0.00	0.10	0.967
Dosinia exoleta	0.00356	0.01122	0.31720	0.00	0.10	0.971
Nucula nucleus	0.00322	0.00988	0.32580	0.10	0.00	0.975

Caulleriella zetlandica	0.00310	0.00971	0.31960	0.00	0.10	0.979
Exogone dispar	0.00310	0.00971	0.31960	0.00	0.10	0.983
Peresiella clymenoides	0.00310	0.00971	0.31960	0.00	0.10	0.987
Glyphohesione klatti	0.00224	0.00690	0.32410	0.00	0.10	0.990
Abra prismatica	0.00224	0.00690	0.32410	0.00	0.10	0.992
Amphiura filiformis	0.00224	0.00690	0.32410	0.00	0.10	0.995
Prionospio caspersi	0.00206	0.00627	0.32870	0.10	0.00	0.998
Nepthys sp	0.00201	0.00611	0.32880	0.10	0.00	1.000

Comparison between Mud and muddy-Sand habitats

	Mean contribution	sd	ratio	M abundance	mS abundance	Cum. sum
Abra nitida	0.09011	0.07673	1.17440	1.64	0.00	0.119
Nematoda	0.08388	0.05206	1.61130	1.93	1.58	0.229
Magelona minuta	0.07940	0.06098	1.30200	1.92	1.46	0.334
Abyssoninoe hibernica	0.05487	0.03253	1.68670	1.19	0.00	0.406
Nucula tenuis	0.02722	0.03427	0.79420	0.67	0.00	0.442
Nucula sulcata	0.02614	0.03678	0.71070	0.50	0.10	0.477
Ophryotrocha sp	0.02574	0.04409	0.58390	0.51	0.10	0.511
Diplocirrus glaucus	0.02458	0.02804	0.87690	0.68	0.10	0.543
Nemertea	0.01800	0.02497	0.72080	0.48	0.10	0.567
Corbula gibba	0.01639	0.03705	0.44240	0.20	0.14	0.588
Scoloplos armiger	0.01228	0.02137	0.57450	0.39	0.10	0.605
Levinsenia gracilis	0.01165	0.02562	0.45460	0.20	0.00	0.620
Aricidea cerrutii	0.01154	0.02464	0.46850	0.24	0.00	0.635
Tubificoides amplivasatus	0.01015	0.02301	0.44090	0.14	0.17	0.649
Magelona indet	0.00928	0.02860	0.32440	0.26	0.00	0.661
Ophiodromus flexuosus	0.00922	0.01924	0.47910	0.20	0.00	0.673
Pholoe baltica	0.00920	0.02276	0.40410	0.20	0.00	0.685

Edwardsiidae	0.00876	0.02132	0.41080	0.20	0.00	0.697
Praxillella affinis	0.00826	0.01745	0.47300	0.39	0.00	0.708
Praxiella sp	0.00826	0.01745	0.47300	0.39	0.00	0.718
Prionospio minuspio	0.00826	0.01745	0.47300	0.39	0.00	0.729
Prionospio sp	0.00826	0.01745	0.47300	0.39	0.00	0.740
Pseudomystides limbata	0.00826	0.01745	0.47300	0.39	0.00	0.751
Pseudomystides sp	0.00826	0.01745	0.47300	0.39	0.00	0.762
Pseudonotomastus southerni	0.00826	0.01745	0.47300	0.39	0.00	0.773
Pseudopolydora pulchra	0.00826	0.01745	0.47300	0.39	0.00	0.784
Scalibregma inflatum	0.00826	0.01745	0.47300	0.39	0.00	0.795
Scolelepis foliosus	0.00826	0.01745	0.47300	0.39	0.00	0.806
Nephtys hystricis	0.00824	0.01803	0.45670	0.00	0.20	0.816
Aricidea suecica	0.00785	0.01698	0.46240	0.22	0.10	0.827
Exogone dispar	0.00747	0.01596	0.46810	0.00	0.20	0.837
Thyasira flexuosa	0.00746	0.02397	0.31120	0.10	0.00	0.846
Prionospio multibranchiata	0.00716	0.02293	0.31220	0.10	0.00	0.856
Mediomastus fragilis	0.00690	0.01553	0.44460	0.20	0.00	0.865
Prionospio fallax	0.00659	0.02097	0.31410	0.10	0.00	0.874
Iphinoe serrata	0.00654	0.02098	0.31170	0.00	0.14	0.882
Harpinia antennaria	0.00582	0.01851	0.31430	0.00	0.14	0.890
Aricidea indet	0.00562	0.01197	0.46960	0.20	0.00	0.897
Ophiuroidea juv	0.00533	0.01647	0.32330	0.00	0.22	0.904
Grania	0.00496	0.01529	0.32440	0.14	0.00	0.911
Apistobranchus tullbergi	0.00473	0.01477	0.32040	0.10	0.00	0.917
Retusa truncatula	0.00473	0.01477	0.32040	0.10	0.00	0.923
Leucon nasica	0.00473	0.01477	0.32040	0.10	0.00	0.930
Notomastus sp	0.00462	0.01483	0.31170	0.00	0.10	0.936
Nephtys incisa	0.00449	0.01397	0.32120	0.10	0.00	0.942
Paradoneis lyra	0.00413	0.01276	0.32330	0.00	0.17	0.947
Paraonidae indet	0.00411	0.01309	0.31430	0.00	0.10	0.953

Amphiura chiajei	0.00411	0.01309	0.31430	0.00	0.10	0.958
Golfingia elongata	0.00411	0.01309	0.31430	0.00	0.10	0.963
Nucula nucleus	0.00351	0.01081	0.32440	0.10	0.00	0.968
Echinocyamus pusillus	0.00337	0.01042	0.32330	0.00	0.14	0.972
Cirrophorus furcatus	0.00238	0.00737	0.32330	0.00	0.10	0.976
Glycinde nordmanii	0.00238	0.00737	0.32330	0.00	0.10	0.979
Magelona filiformis	0.00238	0.00737	0.32330	0.00	0.10	0.982
Syllis sp	0.00238	0.00737	0.32330	0.00	0.10	0.985
Harpinia sp	0.00238	0.00737	0.32330	0.00	0.10	0.988
Urothoe elegans	0.00238	0.00737	0.32330	0.00	0.10	0.991
Aspidosiphon mulleri	0.00238	0.00737	0.32330	0.00	0.10	0.994
Prionospio caspersi	0.00217	0.00662	0.32820	0.10	0.00	0.997
Nepthys sp	0.00212	0.00645	0.32840	0.10	0.00	1.000

<u>Comparison between Mud and sandy-Mud habitats</u>

	Mean contribution	sd	ratio	M abundance	sM abundance	Cum. sum
Magelona minuta	0.07180	0.06607	1.08670	1.92	3.32	0.113
Nematoda	0.06057	0.04171	1.45210	1.93	1.75	0.208
Abra nitida	0.05938	0.05495	1.08070	1.64	0.68	0.302
Abyssoninoe hibernica	0.04065	0.02870	1.41620	1.19	0.20	0.366
Nucula sulcata	0.02530	0.03096	0.81710	0.50	0.37	0.406
Nucula tenuis	0.02300	0.02799	0.82190	0.67	0.00	0.442
Ophryotrocha sp	0.02266	0.03338	0.67890	0.51	0.20	0.478
Diplocirrus glaucus	0.02246	0.02318	0.96890	0.68	0.24	0.513
Nemertea	0.01722	0.02131	0.80820	0.48	0.20	0.540
Corbula gibba	0.01539	0.02735	0.56260	0.20	0.20	0.564
Aricidea cerrutii	0.01184	0.02108	0.56140	0.24	0.10	0.583
Magelona indet	0.01177	0.02676	0.43980	0.26	0.14	0.601

Levinsenia gracilis	0.01081	0.02047	0.52800	0.20	0.10	0.618
Urothoe elegans	0.01056	0.01801	0.58640	0.00	0.30	0.635
Paraonidae indet	0.00956	0.02172	0.44010	0.00	0.20	0.650
Edwardsiidae	0.00878	0.01747	0.50260	0.20	0.10	0.664
Paradoneis lyra	0.00870	0.01847	0.47090	0.00	0.24	0.678
Ophiodromus flexuosus	0.00790	0.01636	0.48290	0.20	0.00	0.690
Praxillella affinis	0.00769	0.01623	0.47380	0.39	0.00	0.702
Praxiella sp	0.00769	0.01623	0.47380	0.39	0.00	0.714
Prionospio minuspio	0.00769	0.01623	0.47380	0.39	0.00	0.726
Prionospio sp	0.00769	0.01623	0.47380	0.39	0.00	0.738
Pseudomystides limbata	0.00769	0.01623	0.47380	0.39	0.00	0.751
Pseudomystides sp	0.00769	0.01623	0.47380	0.39	0.00	0.763
Pseudonotomastus southerni	0.00769	0.01623	0.47380	0.39	0.00	0.775
Pseudopolydora pulchra	0.00769	0.01623	0.47380	0.39	0.00	0.787
Scalibregma inflatum	0.00769	0.01623	0.47380	0.39	0.00	0.799
Scolelepis foliosus	0.00769	0.01623	0.47380	0.39	0.00	0.811
Scoloplos armiger	0.00769	0.01623	0.47380	0.39	0.00	0.823
Pholoe baltica	0.00758	0.01795	0.42240	0.20	0.00	0.835
Aricidea suecica	0.00651	0.01456	0.44730	0.22	0.10	0.845
Mediomastus fragilis	0.00606	0.01334	0.45440	0.20	0.00	0.855
Thyasira flexuosa	0.00582	0.01840	0.31630	0.10	0.00	0.864
Tubificoides amplivasatus	0.00571	0.01770	0.32280	0.14	0.00	0.873
Prionospio multibranchiata	0.00564	0.01779	0.31700	0.10	0.00	0.882
Glycera fallax	0.00547	0.01780	0.30740	0.00	0.10	0.890
Prionospio fallax	0.00529	0.01661	0.31830	0.10	0.00	0.899
Aricidea indet	0.00509	0.01073	0.47440	0.20	0.00	0.907
Aricidea catherinae	0.00449	0.01439	0.31230	0.00	0.10	0.914
Grania	0.00441	0.01354	0.32580	0.14	0.00	0.921
Apistobranchus tullbergi	0.00404	0.01251	0.32280	0.10	0.00	0.927
Retusa truncatula	0.00404	0.01251	0.32280	0.10	0.00	0.934

Leucon nasica	0.00404	0.01251	0.32280	0.10	0.00	0.940
Harpinia antennaria	0.00399	0.01267	0.31490	0.00	0.10	0.946
Nephtys incisa	0.00386	0.01194	0.32340	0.10	0.00	0.952
Amphictene auricoma	0.00353	0.01114	0.31730	0.00	0.10	0.958
Glyphohesione klatti	0.00324	0.01000	0.32380	0.00	0.14	0.963
Nucula nucleus	0.00312	0.00957	0.32580	0.10	0.00	0.968
Ancistrosyllis groenlandica	0.00297	0.00928	0.32030	0.00	0.10	0.973
Exogone dispar	0.00297	0.00928	0.32030	0.00	0.10	0.977
Laubieriellus salzi	0.00297	0.00928	0.32030	0.00	0.10	0.982
Goneplax rhomboides	0.00297	0.00928	0.32030	0.00	0.10	0.987
Exogone hebes	0.00229	0.00707	0.32380	0.00	0.10	0.990
Nephtys hystricis	0.00229	0.00707	0.32380	0.00	0.10	0.994
Prionospio caspersi	0.00202	0.00615	0.32880	0.10	0.00	0.997
Nepthys sp	0.00197	0.00600	0.32890	0.10	0.00	1.000
Magelona minuta	0.07180	0.06607	1.08670	1.92	3.32	0.113
Nematoda	0.06057	0.04171	1.45210	1.93	1.75	0.208
Abra nitida	0.05938	0.05495	1.08070	1.64	0.68	0.302

Comparison between Sand and muddy-Sand habitats

	Mean contribution	sd	ratio	S abundance	mS abundance	Cum. sum
Ophryotrocha sp	0.08906	0.11402	0.78110	1.82	0.10	0.115
Nematoda	0.08834	0.07857	1.12440	0.64	1.58	0.229
Magelona minuta	0.07627	0.09231	0.82630	0.00	1.46	0.328
Grania	0.07059	0.08588	0.82190	0.97	0.00	0.419
Echinocyamus pusillus	0.05146	0.05031	1.02290	0.77	0.14	0.486
Aspidosiphon mulleri	0.03034	0.04163	0.72900	0.44	0.10	0.525
Tubificoides amplivasatus	0.02258	0.04103	0.55050	0.30	0.17	0.554

Corbula gibba	0.02198	0.05341	0.41150	0.10	0.14	0.583
Harpinia antennaria	0.02142	0.05447	0.39320	0.10	0.14	0.610
Paraonidae indet	0.01971	0.02962	0.66520	0.38	0.10	0.636
Abra nitida	0.01860	0.05246	0.35450	0.24	0.00	0.660
Glycera lapidum	0.01834	0.06005	0.30540	0.20	0.00	0.683
Paradoneis lyra	0.01772	0.03759	0.47150	0.20	0.17	0.706
Scoloplos armiger	0.01637	0.03992	0.41010	0.10	0.10	0.727
Exogone hebes	0.01571	0.02565	0.61260	0.38	0.00	0.748
Syllis amica	0.01490	0.05168	0.28830	0.10	0.00	0.767
Exogone dispar	0.01315	0.02335	0.56320	0.10	0.20	0.784
Diplocirrus glaucus	0.01258	0.02478	0.50770	0.27	0.10	0.800
Nephtys hystricis	0.01163	0.02555	0.45520	0.00	0.20	0.815
Iphinoe serrata	0.00950	0.03008	0.31580	0.00	0.14	0.828
Syllis cornuta	0.00936	0.02941	0.31820	0.17	0.00	0.840
Edwardsiidae	0.00890	0.02907	0.30630	0.10	0.00	0.851
Aricidea indet	0.00867	0.02822	0.30710	0.10	0.00	0.862
Golfingia sp	0.00867	0.02822	0.30710	0.10	0.00	0.874
Aonides paucibranchiata	0.00729	0.01552	0.46960	0.20	0.00	0.883
Notomastus sp	0.00672	0.02127	0.31580	0.00	0.10	0.892
Nucula sulcata	0.00672	0.02127	0.31580	0.00	0.10	0.900
Ophiuroidea juv	0.00643	0.01968	0.32670	0.00	0.22	0.909
Amphiura chiajei	0.00571	0.01793	0.31870	0.00	0.10	0.916
Golfingia elongata	0.00571	0.01793	0.31870	0.00	0.10	0.924
Amphictene auricoma	0.00540	0.01698	0.31820	0.10	0.00	0.930
Dosinia exoleta	0.00540	0.01698	0.31820	0.10	0.00	0.937
Aricidea catherinae	0.00496	0.01520	0.32630	0.17	0.00	0.944
Caulleriella zetlandica	0.00443	0.01377	0.32140	0.10	0.00	0.950
Peresiella clymenoides	0.00443	0.01377	0.32140	0.10	0.00	0.955
Aricidea suecica	0.00438	0.01359	0.32250	0.00	0.10	0.961
Nemertea	0.00438	0.01359	0.32250	0.00	0.10	0.967

Cirrophorus furcatus	0.00288	0.00880	0.32670	0.00	0.10	0.970
Glycinde nordmanii	0.00288	0.00880	0.32670	0.00	0.10	0.974
Magelona filiformis	0.00288	0.00880	0.32670	0.00	0.10	0.978
Syllis sp	0.00288	0.00880	0.32670	0.00	0.10	0.982
Harpinia sp	0.00288	0.00880	0.32670	0.00	0.10	0.985
Urothoe elegans	0.00288	0.00880	0.32670	0.00	0.10	0.989
Glyphohesione klatti	0.00286	0.00878	0.32630	0.10	0.00	0.993
Abra prismatica	0.00286	0.00878	0.32630	0.10	0.00	0.996
Amphiura filiformis	0.00286	0.00878	0.32630	0.10	0.00	1.000

Comparison between Sand and sandy-Mud habitats

	Mean contribution	sd	ratio	S abundance	sM abundance	Cum. sum
Magelona minuta	0.16686	0.08552	1.95110	0.00	3.32	0.208
Nematoda	0.08190	0.06695	1.22330	0.64	1.75	0.310
Ophryotrocha sp	0.07251	0.09379	0.77310	1.82	0.20	0.401
Grania	0.05326	0.05962	0.89320	0.97	0.00	0.467
Abra nitida	0.04277	0.05321	0.80370	0.24	0.68	0.520
Echinocyamus pusillus	0.03933	0.03640	1.08070	0.77	0.00	0.569
Paraonidae indet	0.02388	0.03612	0.66130	0.38	0.20	0.599
Aspidosiphon mulleri	0.02333	0.03170	0.73590	0.44	0.00	0.628
Paradoneis lyra	0.01862	0.03065	0.60750	0.20	0.24	0.652
Corbula gibba	0.01740	0.03198	0.54430	0.10	0.20	0.673
Nucula sulcata	0.01692	0.03160	0.53550	0.00	0.37	0.694
Diplocirrus glaucus	0.01567	0.02432	0.64420	0.27	0.24	0.714
Exogone hebes	0.01506	0.02191	0.68700	0.38	0.10	0.733

Tubificoides amplivasatus	0.01444	0.02754	0.52420	0.30	0.00	0.751
Urothoe elegans	0.01426	0.02440	0.58460	0.00	0.30	0.768
Glycera lapidum	0.01354	0.04333	0.31260	0.20	0.00	0.785
Harpinia antennaria	0.01324	0.03253	0.40710	0.10	0.10	0.802
Aricidea catherinae	0.01042	0.02308	0.45140	0.17	0.10	0.815
Nemertea	0.00979	0.02118	0.46200	0.00	0.20	0.827
Syllis amica	0.00924	0.03057	0.30230	0.10	0.00	0.839
Edwardsiidae	0.00880	0.02181	0.40340	0.10	0.10	0.850
Abyssoninoe hibernica	0.00878	0.01993	0.44030	0.00	0.20	0.861
Glycera fallax	0.00862	0.02778	0.31020	0.00	0.10	0.871
Amphictene auricoma	0.00844	0.01873	0.45040	0.10	0.10	0.882
Syllis cornuta	0.00781	0.02433	0.32120	0.17	0.00	0.892
Scoloplos armiger	0.00761	0.02462	0.30920	0.10	0.00	0.901
Exogone dispar	0.00701	0.01538	0.45570	0.10	0.10	0.910
Aricidea indet	0.00650	0.02073	0.31370	0.10	0.00	0.918
Golfingia sp	0.00650	0.02073	0.31370	0.10	0.00	0.926
Aonides paucibranchiata	0.00642	0.01350	0.47540	0.20	0.00	0.934
Glyphohesione klatti	0.00613	0.01340	0.45790	0.10	0.14	0.942
Magelona indet	0.00531	0.01638	0.32430	0.00	0.14	0.948
Aricidea cerrutii	0.00467	0.01453	0.32170	0.00	0.10	0.954
Dosinia exoleta	0.00451	0.01404	0.32120	0.10	0.00	0.960
Caulleriella zetlandica	0.00382	0.01180	0.32350	0.10	0.00	0.964
Peresiella clymenoides	0.00382	0.01180	0.32350	0.10	0.00	0.969
Ancistrosyllis groenlandica	0.00376	0.01158	0.32430	0.00	0.10	0.974
Laubieriellus salzi	0.00376	0.01158	0.32430	0.00	0.10	0.979
Goneplax rhomboides	0.00376	0.01158	0.32430	0.00	0.10	0.983
Aricidea suecica	0.00275	0.00840	0.32700	0.00	0.10	0.987
Levinsenia gracilis	0.00275	0.00840	0.32700	0.00	0.10	0.990
Nephtys hystricis	0.00275	0.00840	0.32700	0.00	0.10	0.994
Abra prismatica	0.00260	0.00795	0.32730	0.10	0.00	0.997

Amphiura filiformis	0.00260	0.00795	0.32730	0.10	0.00	1.000
Magelona minuta	0.16686	0.08552	1.95110	0.00	3.32	0.208
Nematoda	0.08190	0.06695	1.22330	0.64	1.75	0.310

<u>Comparison between Muddy-sand and sandy-Mud habitats</u>

	Mean contribution	sd	ratio	mS abundance	sM abundance	Cum. sum
Magelona minuta	0.17536	0.12079	1.45180	1.46	3.32	0.262
Nematoda	0.11205	0.09065	1.23620	1.58	1.75	0.430
Abra nitida	0.04757	0.06541	0.72720	0.00	0.68	0.501
Corbula gibba	0.02687	0.05240	0.51290	0.14	0.20	0.541
Paraonidae indet	0.02324	0.05311	0.43760	0.10	0.20	0.576
Nucula sulcata	0.02317	0.03905	0.59330	0.10	0.37	0.611
Urothoe elegans	0.01800	0.02944	0.61120	0.10	0.30	0.638
Paradoneis lyra	0.01743	0.03109	0.56050	0.17	0.24	0.664
Ophryotrocha sp	0.01731	0.03218	0.53790	0.10	0.20	0.690
Nemertea	0.01419	0.02666	0.53210	0.10	0.20	0.711
Harpinia antennaria	0.01314	0.02934	0.44780	0.14	0.10	0.731
Nephtys hystricis	0.01289	0.02452	0.52590	0.20	0.10	0.750
Exogone dispar	0.01247	0.02217	0.56260	0.20	0.10	0.769
Glycera fallax	0.01173	0.03943	0.29740	0.00	0.10	0.786
Diplocirrus glaucus	0.01144	0.02046	0.55940	0.10	0.24	0.803
Abyssoninoe hibernica	0.01021	0.02411	0.42370	0.00	0.20	0.818
Iphinoe serrata	0.00893	0.02840	0.31440	0.14	0.00	0.832
Aricidea catherinae	0.00791	0.02554	0.30960	0.00	0.10	0.844
Scoloplos armiger	0.00679	0.02173	0.31250	0.10	0.00	0.854
Aricidea suecica	0.00669	0.01491	0.44830	0.10	0.10	0.864

Notomastus sp	0.00632	0.02009	0.31440	0.10	0.00	0.873
Ophiuroidea juv	0.00625	0.01914	0.32670	0.22	0.00	0.883
Magelona indet	0.00589	0.01827	0.32230	0.00	0.14	0.891
Amphiura chiajei	0.00542	0.01704	0.31790	0.10	0.00	0.900
Golfingia elongata	0.00542	0.01704	0.31790	0.10	0.00	0.908
Amphictene auricoma	0.00535	0.01679	0.31830	0.00	0.10	0.916
Aricidea cerrutii	0.00535	0.01679	0.31830	0.00	0.10	0.924
Tubificoides amplivasatus	0.00484	0.01482	0.32670	0.17	0.00	0.931
Glyphohesione klatti	0.00417	0.01280	0.32610	0.00	0.14	0.937
Ancistrosyllis groenlandica	0.00416	0.01292	0.32230	0.00	0.10	0.943
Laubieriellus salzi	0.00416	0.01292	0.32230	0.00	0.10	0.950
Goneplax rhomboides	0.00416	0.01292	0.32230	0.00	0.10	0.956
Echinocyamus pusillus	0.00395	0.01210	0.32670	0.14	0.00	0.962
Exogone hebes	0.00295	0.00905	0.32610	0.00	0.10	0.966
Levinsenia gracilis	0.00295	0.00905	0.32610	0.00	0.10	0.971
Edwardsiidae	0.00295	0.00905	0.32610	0.00	0.10	0.975
Cirrophorus furcatus	0.00280	0.00856	0.32670	0.10	0.00	0.979
Glycinde nordmanii	0.00280	0.00856	0.32670	0.10	0.00	0.983
Magelona filiformis	0.00280	0.00856	0.32670	0.10	0.00	0.988
Syllis sp	0.00280	0.00856	0.32670	0.10	0.00	0.992
Harpinia sp	0.00280	0.00856	0.32670	0.10	0.00	0.996
Aspidosiphon mulleri	0.00280	0.00856	0.32670	0.10	0.00	1.000

Figure S5: No significant effects were found of sediment type and climate regime on a) maximum particle reworking depth (mean $^{f\text{-}SPI}L_{max}$ ± s.e, cm), b) surface boundary roughness (cm) and c) burrow ventilation (ΔBr^-). Climate regimes are indicated by colour (black = Ambient: 11°C, 380ppm [CO_2]; grey = Future: 15°C, 1000ppm [CO_2]) and sediment types are: M = mud, sM = sandy mud, mS = muddy sand, S = sand.

a)

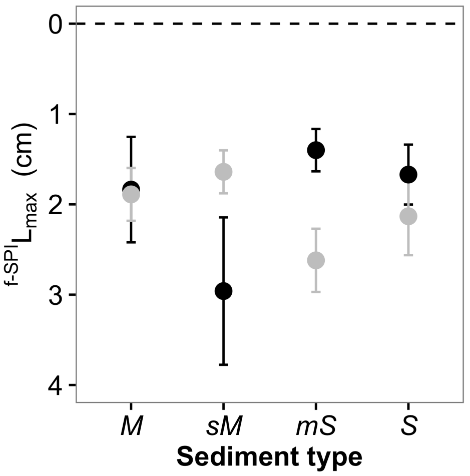

b)

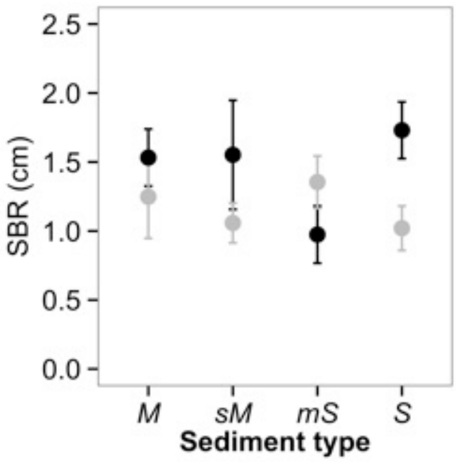

c)

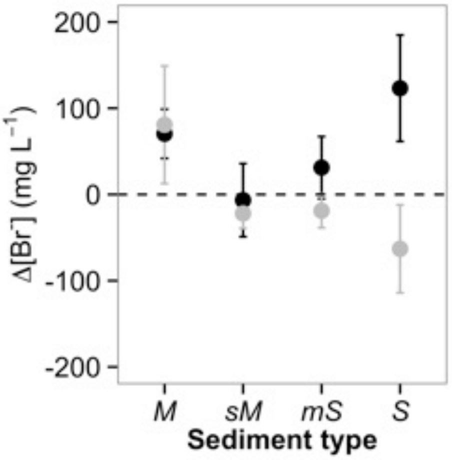

Figure S6: Average species richness (± s.e) per functional group in mud (M), muddy Sand (mS), sandy mud (sM) and sand (S) under the a) ambient (11°C, 380ppm [CO_2]) and b) future (15°C, 1000ppm [CO_2]) climate regime. 2 = surficial modifier, 3 = upward/downward conveyor, 4 = biodiffuser.

a)

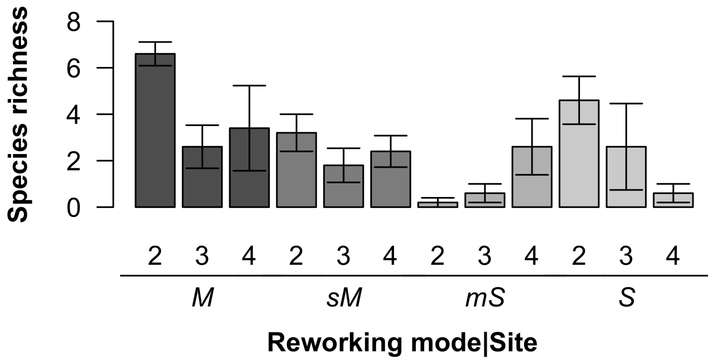

b)

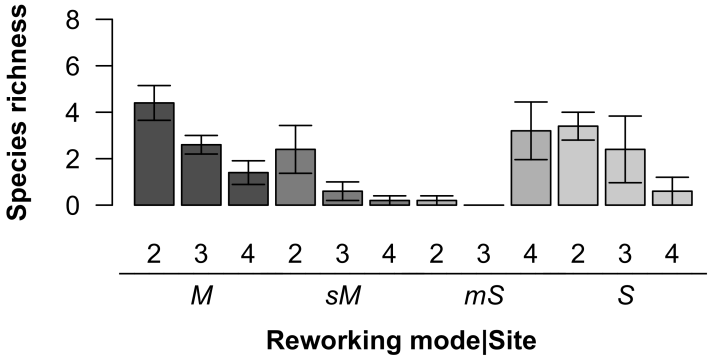

ENDS
